# Treatment Effects of Different Therapeutic Strategies and Development of a Prognostic Prediction Model for Early Gastric Cancer: A Population-Based and Multicenter Study

**DOI:** 10.3390/cancers18213488

**Published:** 2026-10-29

**Authors:** Ruiying Jing, Shimei Sun, Tian Tian, Zhiyu Xiang, Wei Su, Hunter Wang, Michael J. Bourke, Shengli Lin, Yaoyao Gong

**Affiliations:** 1 Department of Gastroenterology, Jiangsu Province Hospital (the First Affiliated Hospital of Nanjing Medical University), Nanjing 210029, China; jry0924@stu.njmu.edu.cn (R.J.); ttian@stu.njmu.edu.cn (T.T.); 2 Department of Gastroenterology, Hospital of Sihong, No. 31, Jiankang Road, Sihong 223900, China; jmj007@126.com; 3 Endoscopy Center and Endoscopy Research Institute, Zhongshan Hospital, Fudan University & Shanghai Collaborative Innovation Center of Endoscopy, Shanghai 200032, China; 25211210144@m.fudan.edu.cn (Z.X.); su.wei1@zs-hospital.sh.cn (W.S.); 4 Department of Gastroenterology and Hepatology, Westmead Hospital, Sydney 2145, Australia; hunter.wang@health.nsw.gov.au (H.W.); michael@citywestgastro.com.au (M.J.B.); 5 Department of Gastroenterology, Chongqing Hospital of Jiangsu Province Hospital, Chongqing 401420, China

**Keywords:** early gastric cancer, prediction model, survival prognosis

## Abstract

**Background**: Early gastric cancer (EGC) lacks a predictive tool integrating endoscopic therapy (ET), gastrectomy (G), and chemoradiotherapy (CRT) to support individualized risk stratification. This study compared cancer-specific survival (CSS) and overall survival (OS) across the four treatment strategies and developed and externally validated a CSS prediction model incorporating oncologic characteristics and treatment strategy (presented as a nomogram and online tool). **Methods**: In a U.S. cohort (2017–2021), 1766 patients with EGC were classified into four treatments groups (ET, ET + CRT, G, G + CRT); an independent multicenter cohort (*n* = 1333) provided external validation. Kaplan–Meier curves, log-rank tests, and Cox regression assessed unadjusted differences, with confounding balanced by stabilized inverse probability of treatment weighting (sIPTW). Predictors were selected by univariable Cox, LASSO, Boruta, and best-subset regression; performance was evaluated by C-index, calibration curves, and decision curve analysis. **Results**: After sIPTW adjustment, CSS did not differ significantly between ET and G (*p* = 0.856), whereas G was associated with better CSS than G + CRT (*p* < 0.05). In exploratory analyses of node-positive patients, G + CRT was associated with lower cancer-specific mortality than G alone (*p* < 0.05). The CSS prediction model was validated internally and externally (AUCs for 1-, 3-, and 5-year CSS: 0.769, 0.737, 0.717 internally; 0.943, 0.890, 0.872 externally). **Conclusions**: This is the first externally validated EGC CSS model integrating all four treatments. For eligible patients, ET was associated with survival comparable to G and superior to G + CRT; among node-positive patients, G + CRT was associated with lower mortality than G alone. The model supports individualized prediction and treatment decisions.

## Simple Summary

Early gastric cancer can be treated using endoscopic procedures, surgery, or additional chemoradiotherapy, but long-term outcomes vary across patients and treatment strategies. We analyzed data from 1766 patients in a population-based cancer registry and 1333 patients from an independent multicenter cohort to compare survival outcomes and develop a model for predicting cancer-specific survival. After accounting for measured differences between treatment groups, endoscopic therapy and gastrectomy showed no statistically significant difference in cancer-specific survival in the population-based cohort. Among patients with lymph node metastasis, gastrectomy combined with chemoradiotherapy was associated with lower cancer-specific mortality than gastrectomy alone. The prediction model showed good discrimination in an independent clinical cohort and was made available as an online calculator. These findings provide additional evidence on treatment-related survival outcomes and a tool for individualized prognosis assessment in early gastric cancer.

## 1. Introduction

Gastric cancer is among the most common malignancies worldwide. According to GLOBOCAN 2022, approximately 968,350 new gastric cancer cases and 659,853 deaths occurred globally, making it one of the five most common malignancies worldwide and one of the four leading causes of cancer-related death [1].

Early gastric cancer (EGC) is defined as carcinoma with invasion limited to the mucosal or submucosal layer, regardless of lymph node metastasis; the overall 5-year survival rate of EGC can exceed 90% [2]. Current guidelines recommend that treatment strategies for EGC be primarily determined by preoperative assessment of tumor invasion depth and lymph node metastasis risk. The National Comprehensive Cancer Network (NCCN) guidelines [3,4] recommend endoscopic submucosal dissection (ESD) as the standard treatment for clinical T stage 1a (cT1a) tumors (invading the lamina propria or muscularis mucosae) and selected superficial cT1b tumors (submucosal invasion ≤ 500 μm) when the risk of lymph node metastasis is very low. Gastrectomy remains an option for eligible surgical candidates, whereas it is recommended for patients with deep cT1b tumors (submucosal invasion > 500 μm). For non-curative endoscopic resection, observation or additional gastrectomy is performed as appropriate. Following gastrectomy, patients undergo observation or adjuvant chemoradiotherapy (CRT based on postoperative pathology and lymph node status [3,4].

In addition to histological type and the tumor (T) and regional lymph node (N) categories of the tumor–node–metastasis (TNM) classification, the prognosis of patients with EGC may also be influenced by age, race, treatment modality, the number of regional lymph nodes examined (RNE), the diagnosis-to-treatment time (DTTT), and various other factors. Several models have been developed to predict prognosis or lymph node metastasis in EGC. For example, Cheng et al. used the Surveillance, Epidemiology, and End Results (SEER) database of the U.S. National Cancer Institute to stratify cancer-specific risk in elderly EGC patients and constructed a prediction model [5]. Zheng et al. used the SEER database to build a model for preoperative prediction of lymph node metastasis risk in elderly EGC patients [6]. However, the existing EGC prognostic prediction models [5,6,7,8] have not yet integrated demographic information, RNE, DTTT, and other clinical factors together with the four different treatment strategies.

To address these gaps, this study used the SEER database to evaluate the survival outcomes of EGC patients receiving different treatments. Subsequently, a user-friendly prediction model was developed and externally validated in an independent multicenter cohort, aiming to provide a quantitative tool for individualized risk stratification and clinical decision-making in EGC. This study was reported in accordance with the 2024 Transparent Reporting of a multivariable prediction model for Individual Prognosis Or Diagnosis–Artificial Intelligence (TRIPOD + AI) statement.

## 2. Materials and Methods

### 2.1. Study Design

**Figure 1:**
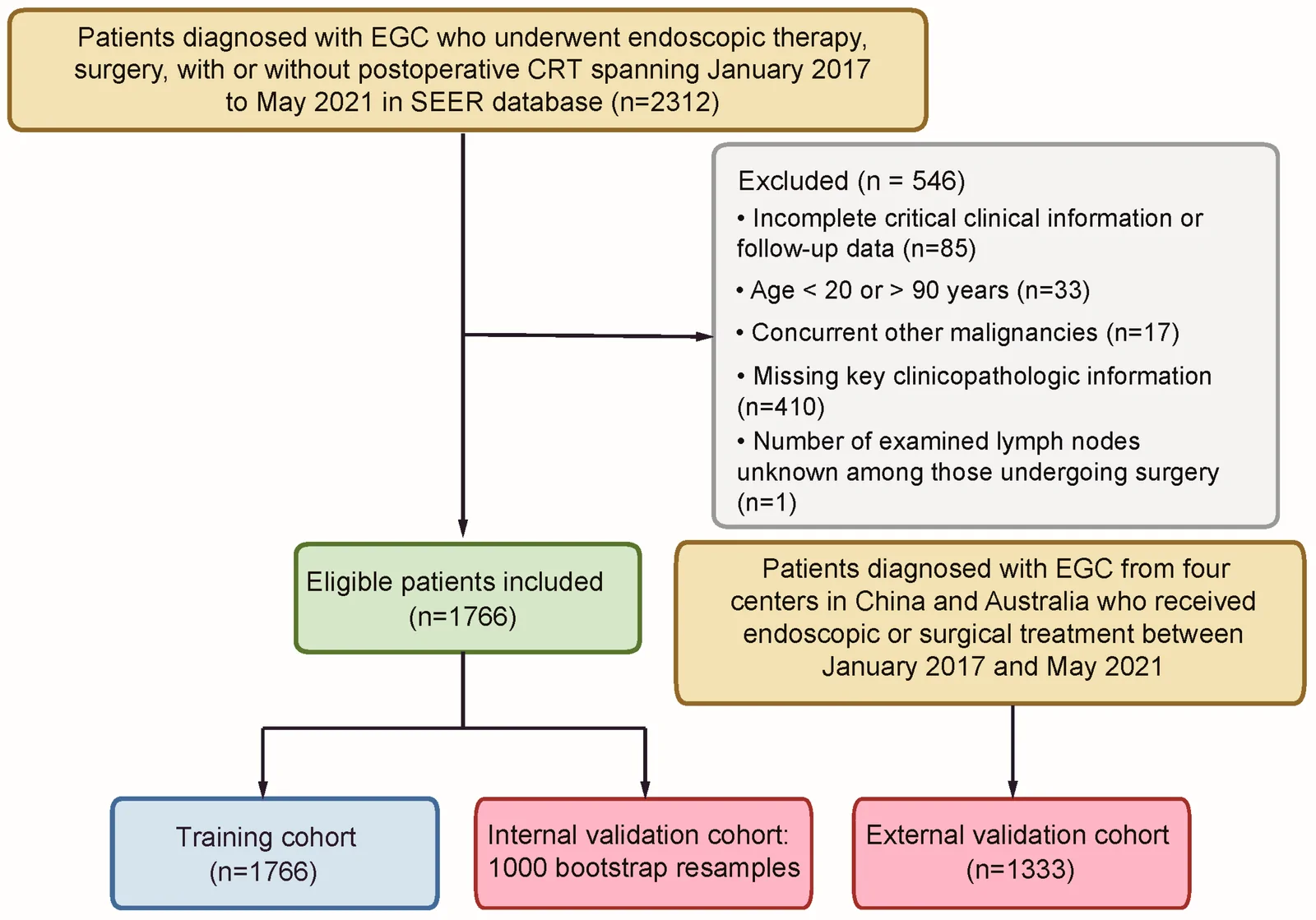
Patient selection flow diagram for the SEER development cohort and the four-center external validation cohort. SEER, Surveillance, Epidemiology, and End Results; EGC, early gastric cancer; CRT, chemoradiotherapy.

The development cohort was extracted from the SEER database (https://seer.cancer.gov/), a U.S. population-based cancer registry maintained by the National Cancer Institute, using SEER*Stat software (version 9.0.42.0). Clinical data of patients diagnosed with early gastric cancer and receiving relevant treatment between January 2017 and May 2021 were collected. The external validation data comprised patients from the EGC databases of four centers—Jiangsu Province Hospital and the First Affiliated Hospital of Nanjing Medical University; Zhongshan Hospital, Fudan University; Westmead Hospital, University of Sydney; and Hospital of Sihong. The inclusion periods were similar to those of the development cohort, and no patients were included in both cohorts. The overall workflow is shown in Figure 1.

### 2.2. Patients

The diagnosis of early gastric cancer was determined according to the International Classification of Diseases for Oncology (ICD-O-3) coding and SEER database pathology coding.

Patients were eligible for inclusion if they had pathologically confirmed EGC registered in the SEER database between January 2017 and May 2021, had received endoscopic therapy or gastrectomy with or without chemoradiotherapy, and had the required clinical and follow-up information available. Patients were excluded if they had an unknown cause of death or survival time; were missing essential follow-up data; were younger than 20 years or older than 90 years; had concurrent non-gastric malignant tumors; had missing essential clinicopathological information; or had an unknown number of lymph nodes examined following gastrectomy.

### 2.3. Study Variables and Outcomes

The variables extracted from the SEER database were as follows: age, sex, race, marital status, primary tumor site, histological type, tumor size, T stage, N stage, radiotherapy administration, chemotherapy administration, RNE, and DTTT. Follow-up data included detailed information such as survival status, survival time, and cause of death. T categories were determined according to pathological staging, whereas N categories were based on clinical staging for patients receiving endoscopic therapy and pathological staging for those undergoing gastrectomy. T and N categories were classified according to the 8th edition of the American Joint Committee on Cancer (AJCC) staging system [9]. Patients with regional lymph node stage N1, N2, or N3 were defined as node-positive. In the SEER database, chemotherapy was recorded as a binary variable, and specific regimen information could not be obtained. The CRT category included patients who received chemotherapy, radiotherapy, or both. SEER*Stat software was used for data extraction, and two investigators independently performed data sorting and verification to ensure accuracy. For missing data, cases were screened and processed according to the pattern of missingness, excluding those with missing key information. For the external validation cohort, additional information was collected, including surgical margin status (whether R0 resection was achieved) and specific chemotherapy regimens, because these data were unavailable in the SEER database. All data were anonymized.

According to ICD-O-3, histological types were classified into three categories: adenocarcinoma (8140–8147, 8210–8211, 8260–8263), signet-ring cell carcinoma (8490), and others (8480 and 8481). In the SEER database, the procedure codes for endoscopic therapy ranged from A100–A140 and A200–A270 (the former represent local tumor-destructive procedures without pathological specimen submission, such as photodynamic therapy, electrocautery, cryotherapy, and laser ablation; the latter represent local tumor resection with specimen submission); the procedure codes for gastrectomy were A300–A800, representing various gastrectomy procedures. The primary endpoints were cancer-specific survival (CSS) and overall survival (OS): CSS was defined as the time from gastric cancer diagnosis to death from gastric cancer-related causes; OS was defined as the time from gastric cancer diagnosis to death from any cause or last follow-up.

### 2.4. Statistical Analysis

All statistical analyses were performed using Python (version 3.13.5). A two-sided *p* < 0.05 was considered statistically significant. Continuous variables were described as mean ± standard deviation or median (interquartile range) according to normality test results and compared between groups using the t-test or Mann–Whitney U test; categorical variables were described as counts (%) and compared using the χ^2^ test or Fisher’s exact test.

In the development cohort, Kaplan–Meier analysis [10] and the log-rank test [11] were used to compare CSS and OS across treatment groups, and univariable Cox proportional hazards models [12] were constructed to calculate hazard ratios (HRs) and 95% confidence intervals (CIs). To address treatment-selection bias arising from non-random treatment allocation, stabilized inverse probability of treatment weights (sIPTW) were derived from propensity scores [13,14], and standardized mean differences (SMD) were used to assess covariate balance after weighting [15], with these Cox analyses repeated in the weighted sample. This workflow was replicated in the external validation cohort to assess reproducibility, and univariable and multivariable Cox proportional hazards regression analyses were performed. The characteristics of patients receiving chemoradiotherapy in the external validation cohort were summarized descriptively, and regimen frequencies were counted by treatment modality. Node-positive patients were divided into N1, N2, and N3 subgroups, and subgroup survival analyses were performed in the development and external validation cohorts, respectively; Monte Carlo exact tests were used to compare regimen distributions across N stages among patients receiving G + CRT.

For CSS prediction, initial variable screening was performed using univariable Cox regression, followed by predictor selection using LASSO regression [16], the Boruta algorithm [17], and best-subset regression (BSR) [18]. The final predictors were then included in a multivariable Cox model. The CSS prediction model was developed using the original, unweighted SEER cohort. Internal validation used 1000 bootstrap resamples [19], and external validation used an independent dataset. The concordance index (C-index) was used to assess overall model discrimination, and time-dependent receiver operating characteristic (ROC) curves and corresponding areas under the curve (AUCs) were used to assess discrimination at 1, 3, and 5 years [19,20]. Calibration curves at 1, 3, and 5 years were used to evaluate the consistency between predicted and observed probabilities. Decision curve analysis (DCA) was used to evaluate the clinical utility of the model [21]. In addition, a sensitivity analysis of the CSS prediction model was performed after excluding non-R0 cases.

## 3. Results

### 3.1. Patient Characteristics

A total of 1766 patients diagnosed with early gastric cancer were included in this study. Their baseline characteristics—including demographic, pathological, and surgical information—by treatment modality are shown in Table 1. Among them, there were 444 cases in the endoscopic therapy (ET) group, 44 in the endoscopic therapy + CRT (ET + CRT) group, 995 in the gastrectomy (G) group, and 283 in the gastrectomy + CRT (G + CRT) group.

### 3.2. Comparison of Unadjusted Survival Outcomes

Compared with G, ET showed superior long-term CSS (HR = 0.58, 95% CI 0.35–0.98, log-rank *p* = 0.040), while the difference in OS was not statistically significant (HR = 1.30, 95% CI 0.99–1.69, log-rank *p* = 0.053). Compared with ET alone, ET + CRT had lower long-term survival (CSS: HR = 6.31, 95% CI 2.98–13.36, log-rank *p* < 0.001; OS: HR = 2.08, 95% CI 1.23–3.50, log-rank *p* = 0.005). Compared with G, ET + CRT also had lower long-term survival (CSS: HR = 3.69, 95% CI 1.95–6.96, log-rank *p* < 0.001; OS: HR = 2.78, 95% CI 1.68–4.58, log-rank *p* < 0.001). Compared with the G group, the G + CRT group had an increased risk of cancer-specific death (HR = 1.60, 95% CI 1.05–2.43, log-rank *p* = 0.026), while the OS difference was not statistically significant (HR = 1.26, 95% CI 0.92–1.72, log-rank *p* = 0.143). Finally, compared with the G + CRT group, the ET + CRT group had lower long-term survival (CSS: HR = 2.36, 95% CI 1.19–4.70, log-rank *p* = 0.011; OS: HR = 2.26, 95% CI 1.31–3.90, log-rank *p* = 0.003). All Kaplan–Meier curves presenting the above CSS and OS results are shown in Figures S1 and S2, and the corresponding 5-year CSS and OS rates by treatment are listed in Table S1.

### 3.3. Comparison of Survival Outcomes Based on sIPTW Adjustment

**Figure 2:**
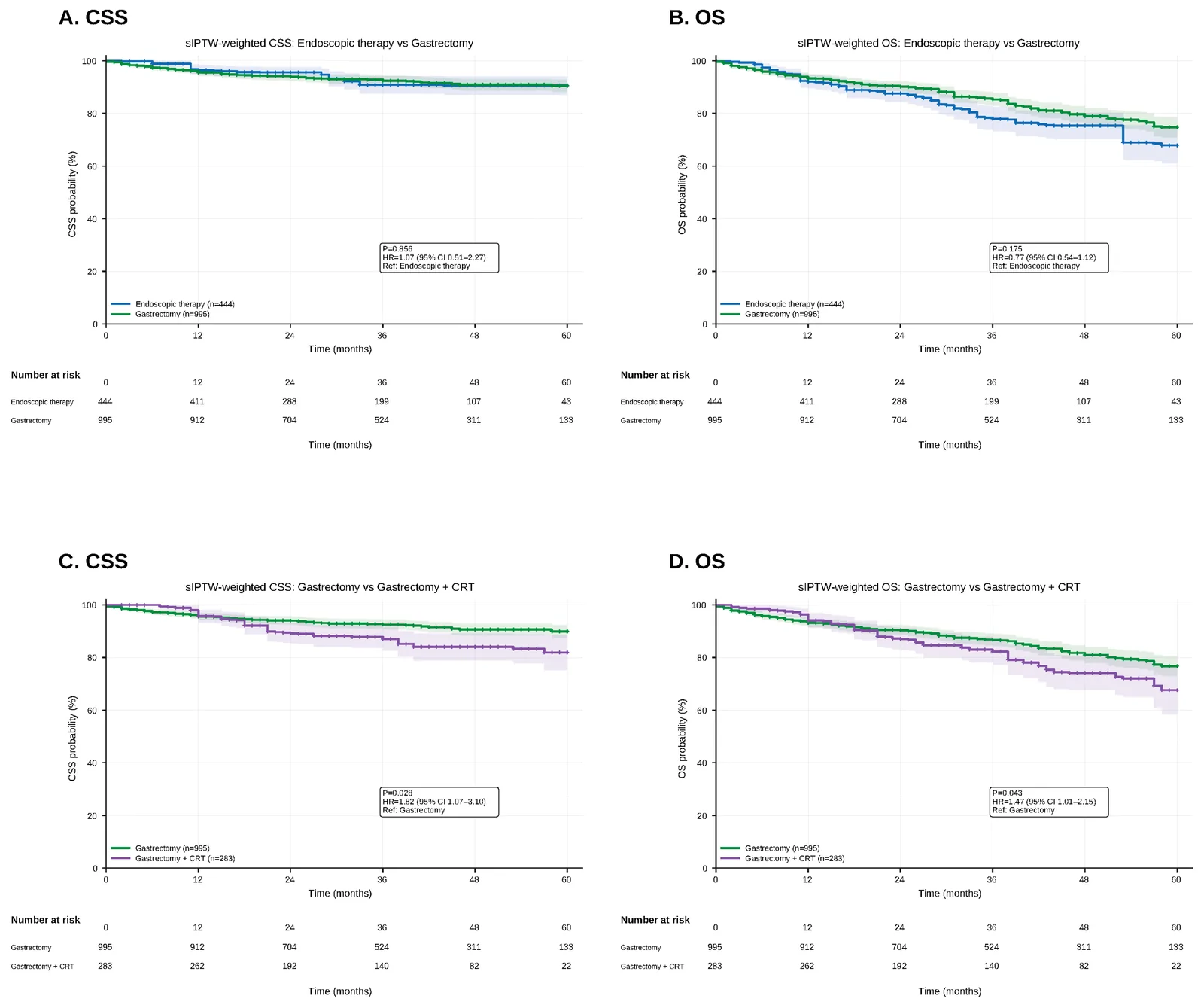
After adjustment using sIPTW, Kaplan–Meier curves were used to compare cancer-specific survival (CSS) and overall survival (OS) outcomes among different treatments for early gastric cancer. Panels show ET versus G for CSS (**A**) and OS (**B**), and G + CRT versus G for CSS (**C**) and OS (**D**). sIPTW, stabilized inverse probability of treatment weighting; CSS, cancer-specific survival; OS, overall survival; CRT, chemoradiotherapy.

After sIPTW adjustment, the survival curves showed that, compared with the ET group, the G group had a cancer-specific death risk HR of 1.07 (95% CI 0.51–2.27, *p* = 0.856) and an all-cause death risk HR of 0.77 (95% CI 0.54–1.12, *p* = 0.175); the two groups showed no statistically significant difference in CSS or OS (Figure 2A,B), and covariate balance data are shown in Tables S2 and S3.

Compared with G alone, the G + CRT group had a higher risk of cancer-specific death (HR = 1.82, 95% CI 1.07–3.10, *p* = 0.028) and also a higher risk of all-cause death (HR = 1.47, 95% CI 1.01–2.15, *p* = 0.043); both the CSS and OS differences were statistically significant (Figure 2C,D), and covariate balance data are shown in Tables S3 and S4.

### 3.4. Comparison of Survival Outcomes in the External Cohort

The 1333 EGC patients from the databases of four centers constituted an external cohort. The baseline characteristics of this external dataset are summarized in Table S5. Among the 1333 patients, 1306 (98.0%) achieved R0 resection, while 27 patients (2.0%) did not achieve R0 resection.

Kaplan–Meier estimates showed that in the external validation cohort the ET group had the highest 5-year CSS and 5-year OS rates, while the ET + CRT group had the lowest (Table S6). Compared with G, ET showed superior results in both CSS and OS (CSS: HR = 0.33, 95% CI 0.13–0.82, *p* = 0.012; OS: HR = 0.53, 95% CI 0.33–0.86, *p* = 0.008). The OS difference between the G + CRT and ET groups was more pronounced (HR = 3.22, 95% CI 1.85–5.61, *p* < 0.001), and CSS was further reduced (HR = 6.93, 95% CI 2.66–18.04, *p* < 0.001). Compared with ET, the ET + CRT group had higher risks of both cancer-specific and all-cause death (CSS: HR = 30.56, 95% CI 10.26–91.07, *p* < 0.001; OS: HR = 9.84, 95% CI 4.57–21.18, *p* < 0.001), but because this subgroup had a small sample size (*n* = 22), the result is exploratory. Between the two surgical modalities, the G + CRT group had a higher risk of cancer-specific death than gastrectomy alone (HR = 2.33, 95% CI 1.18–4.58, *p* = 0.012) and also a higher risk of all-cause death (HR = 1.73, 95% CI 1.08–2.76, *p* = 0.021). The above results are shown in Figures S3 and S4.

After sIPTW adjustment, the G group had higher estimated hazards of cancer-specific and all-cause death than the ET group (CSS: HR = 2.29, 95% CI 0.81–6.46, *p* = 0.117; OS: HR = 2.08, 95% CI 1.18–3.68, *p* = 0.012), with the OS difference being statistically significant. The G + CRT group had higher cancer-specific and all-cause death risks than G alone (CSS: HR = 1.85, 95% CI 0.89–3.85, *p* = 0.099; OS: HR = 1.50, 95% CI 0.85–2.64, *p* = 0.158); the two groups showed no statistically significant difference in CSS or OS (Figure S5). Covariate balance data are shown in Tables S7–S9.

Among the 203 patients who received CRT, 22 received ET + CRT and 181 received G + CRT (Table S10). The most commonly used regimens were S-1 (tegafur, gimeracil, and oteracil potassium; 82 patients, 40.4%), SOX (S-1 plus oxaliplatin; 53 patients, 26.1%), and XELOX (capecitabine plus oxaliplatin; 35 patients, 17.2%), followed by TS (paclitaxel plus S-1; 17 patients, 8.4%), DOS (docetaxel, oxaliplatin, and S-1; 8 patients, 3.9%), FOLFOX (folinic acid, fluorouracil, and oxaliplatin; 3 patients, 1.5%), DS (docetaxel plus S-1; 3 patients, 1.5%), and CAP (capecitabine; 2 patients, 1.0%). There were no statistically significant differences in 5-year overall survival or cancer-specific survival among S-1, SOX, XELOX, and other regimens (OS: log-rank *p* = 0.116; CSS: log-rank *p* = 0.622) (Table S11).

### 3.5. Factors Associated with CSS and OS in the External Cohort

In the multivariable Cox analysis for CSS, age, T stage, N stage, and treatment modality were significantly associated with cancer-specific mortality. After adjustment, tumor size was not independently associated with CSS. Compared with R0 resection, non-R0 resection was associated with an increased risk of cancer-specific death (HR = 4.350, 95% CI 1.563–12.110; univariable analysis *p* = 0.020), although the overall effect of resection status was borderline (multivariable analysis *p* = 0.050) (Table S12, Figure S6A).

In the multivariable Cox analysis, age, T stage, N stage, and treatment modality remained significantly associated with OS, whereas tumor size and RNE showed no independent association with OS (Table S13, Figure S6B).

### 3.6. Subgroup Analysis of Node-Positive Patients

In the node-positive subgroup, the development cohort included 220 patients, and the external validation cohort included 144 patients; baseline characteristics are shown in Tables S14–S16. Table S17 presents the 5-year OS and CSS rates for patients with different N stages; as N stage increased, the 5-year CSS and OS rates in both cohorts showed an overall decline.

Regarding the effect of N stage on prognosis, in the development cohort, patients with N2 disease had a significantly higher risk of cancer-specific death than patients with N1 disease (HR = 2.25, 95% CI 1.07–4.72; log-rank *p* = 0.027, Table S18, Figure S7). In the external validation cohort, patients with N3 disease had a significantly higher risk of cancer-specific death than patients with N1 disease (HR = 3.39, 95% CI 1.25–9.18; log-rank *p* = 0.010, Figure S8).

In the node-positive subgroup, compared with G alone, G + CRT significantly reduced the risk of cancer-specific death (HR = 0.46, 95% CI 0.22–0.93; log-rank *p* = 0.026) and all-cause death (HR = 0.40, 95% CI 0.23–0.70; log-rank *p* < 0.001) in the development cohort. In the external validation cohort, G + CRT also significantly reduced the risk of cancer-specific death (HR = 0.40, 95% CI 0.17–0.96; log-rank *p* = 0.033), while the reduction in all-cause death did not reach statistical significance (HR = 0.61, 95% CI 0.28–1.31; log-rank *p* = 0.200) (Figure S9).

Among the 108 node-positive patients who received G + CRT in the external validation cohort, 33 received the S-1 regimen, 33 received SOX, 19 received XELOX, and 23 received other regimens. The regimen distribution showed a non-significant trend of variation across N stages (*p* = 0.075) (Table S19). The SOX regimen was most commonly used in N2 patients (44.1%), while the S-1 regimen was most common in N3 patients (50.0%). During follow-up, 24 deaths occurred among these 108 node-positive patients, of which 14 were cancer-specific. The 5-year overall survival rates for S-1, SOX, XELOX, and other regimens were 78.8%, 84.8%, 63.2%, and 78.3%, respectively; the corresponding 5-year cancer-specific survival rates were 87.5%, 90.6%, 83.2%, and 82.6%, respectively, with no statistically significant differences among regimens (OS: log-rank *p* = 0.313; CSS: log-rank *p* = 0.771) (Table S20).

### 3.7. Development of the Prediction Model

The training cohort comprised 1766 patients with EGC from the SEER database, and an additional 1333 patients from the multicenter cohort formed the external validation set, totaling 3099 patients.

Univariable Cox regression analysis showed that sex, age, race, tumor size, T stage, N stage, AJCC stage, treatment, and RNE were significantly associated with CSS (*p* < 0.05, Table S21); a candidate set of 10 variables was carried forward for selection (Figure S10, Table S22). On this basis, three complementary strategies were used to screen the final predictors: LASSO regression (10-fold cross-validation with the conservative α_1se criterion; Figure S10A,B), Boruta variable-importance analysis (Figure S10C), and Akaike information criterion (AIC)-based best-subset regression (BSR; Table S22). Integrating the above results, the final multivariable Cox proportional hazards regression model included seven variables: age, sex, race, T stage, N stage, RNE, and treatment (Table S23). Among these, older age, other race, higher T stage, higher N stage, an insufficient number of examined lymph nodes, and endoscopic therapy plus chemoradiotherapy were all independent risk factors for EGC CSS (Table S23, Figure S10D).

**Figure 3:**
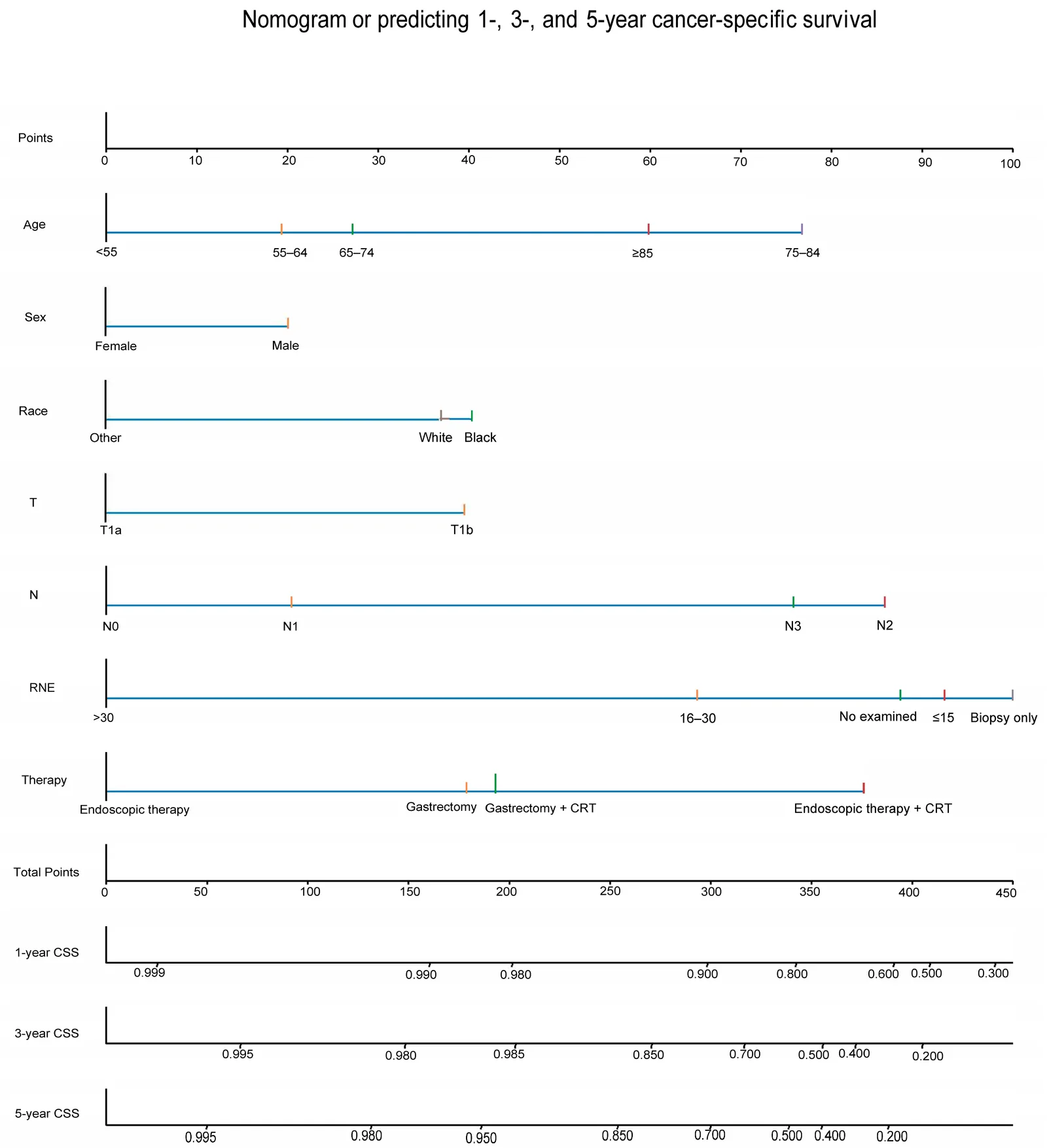
Nomogram for predicting 1-, 3-, and 5-year cancer-specific survival (CSS) in patients with early gastric cancer. The nomogram assigns points to seven variables: age, sex, race, T stage, N stage, number of regional lymph nodes examined (RNE), and treatment modality. The points assigned to each variable are summed, and the total score corresponds to the predicted probabilities of 1-, 3-, and 5-year CSS. CSS, cancer-specific survival; CRT, chemoradiotherapy; EGC, early gastric cancer; RNE, number of regional lymph nodes examined; T, tumor stage; N, node stage.

Based on the variables included in the multivariable Cox proportional hazards regression model—age, sex, race, T stage, N stage, RNE, and treatment modality—a nomogram predicting CSS in gastric cancer patients was constructed (Figure 3); a complementary online interactive prediction tool was also developed (see below).

### 3.8. Predictive Performance of the Model

**Figure 4:**
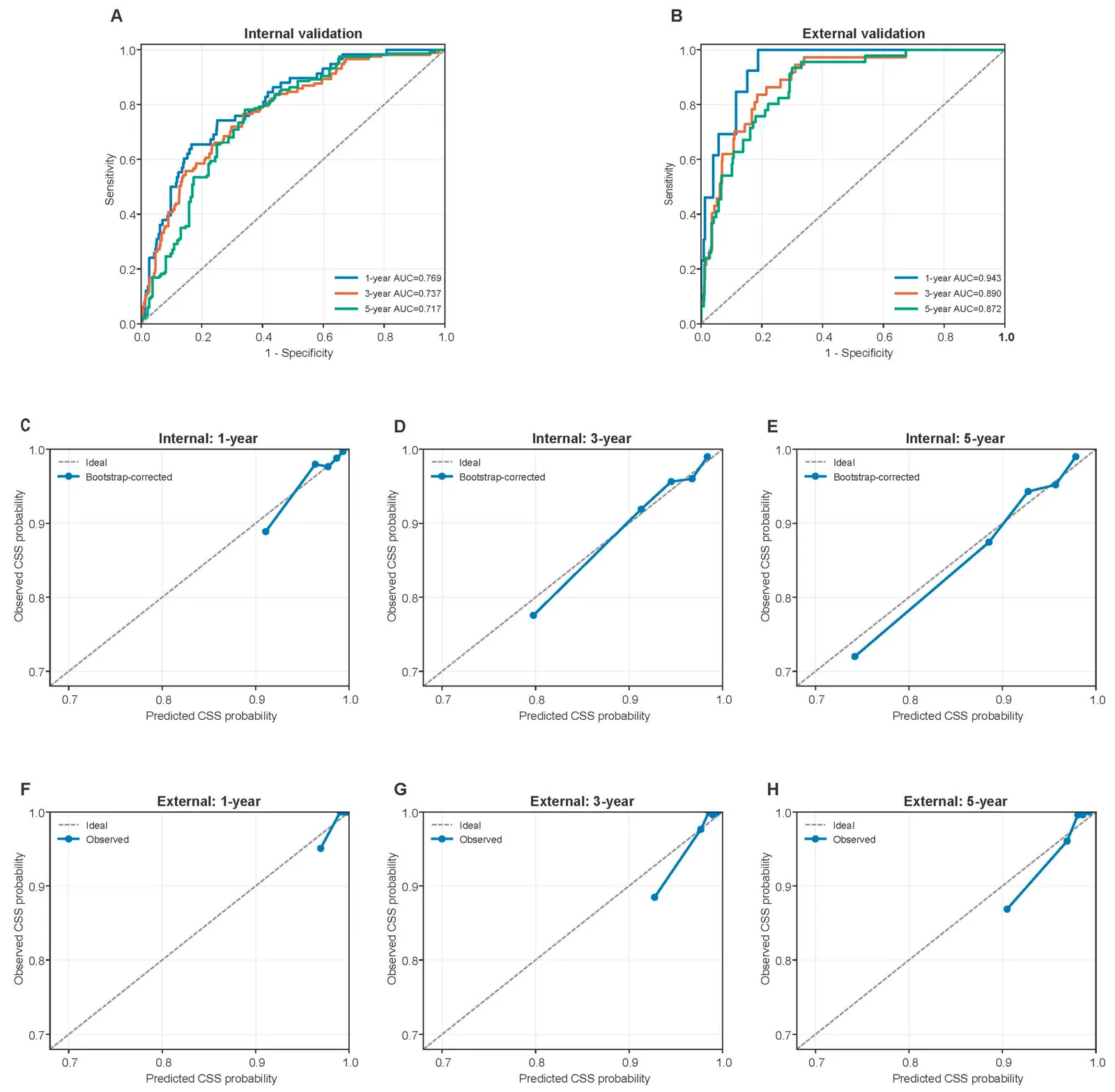
Discrimination and calibration of the nomogram for predicting cancer-specific survival (CSS) in early gastric cancer. (**A**) Time-dependent ROC curves for 1-, 3-, and 5-year CSS in the internal validation cohort; AUCs were 0.769, 0.737, and 0.717, respectively. (**B**) Time-dependent ROC curves for 1-, 3-, and 5-year CSS in the external validation cohort; AUCs were 0.943, 0.890, and 0.872, respectively. (**C**–**E**) Calibration curves for 1-, 3-, and 5-year CSS in the internal validation cohort ((**C**), 1-year; (**D**), 3-year; (**E**), 5-year), comparing predicted versus observed survival probabilities; bootstrap-corrected curves lie close to the ideal reference line. (**F**–**H**) Calibration curves for 1-, 3-, and 5-year CSS in the external validation cohort ((**F**), 1-year; (**G**), 3-year; (**H**), 5-year). CSS, cancer-specific survival; ROC, receiver operating characteristic; AUC, area under the curve.

To evaluate the predictive accuracy of the nomogram, we calculated the C-index. The training cohort C-index was 0.767 (95% CI: 0.726–0.806), the internal validation cohort (bootstrap-corrected) was 0.735 (95% CI: 0.727–0.761), and the external validation cohort was 0.861 (95% CI: 0.815–0.903), indicating good discriminative ability. In addition, ROC curve analysis was performed, and time-dependent AUCs were calculated: the 1-, 3-, and 5-year CSS AUCs were 0.769, 0.737, and 0.717 in the internal validation cohort (Figure 4A) and 0.943, 0.890, and 0.872 in the external validation cohort (Figure 4B), indicating good discrimination. Calibration curves were then drawn to evaluate the consistency between predicted and actual CSS (*x*-axis: predicted survival probability; *y*-axis: observed survival probability): the internal validation 1-, 3-, and 5-year calibration curves (Figure 4C–E) closely followed the ideal reference line; the external validation calibration curves (Figure 4F–H) approximated the observed values at the population average, although some deviation in individual risk gradient was noted, suggesting a slight decrease in long-term predictive accuracy. Decision curve analysis (internal validation: Figure S11A–C; external validation: Figure S11D–F) showed a net clinical benefit for the model at all evaluated time points. In a sensitivity analysis restricted to R0-resected patients, the model C-index remained 0.870 (95% CI: 0.825–0.910), indicating that excluding a small number of non-R0 cases did not materially alter the model’s discriminative ability. In summary, the nomogram showed satisfactory accuracy for CSS prediction.

### 3.9. Web-Based Prediction Tool

In addition to the nomogram, this study simultaneously developed an online interactive prediction model (web calculator). Users can access the https://019fcd9d-b0d2-d48b-ac6a-b5671e8dfb17.share.connect.posit.cloud/ (accessed on 22 October 2026) interactive visualization online. By entering the seven variables above, real-time 1-, 3-, and 5-year CSS predicted probabilities are obtained, facilitating individualized clinical application.

## 4. Discussion

Based on 1766 EGC patients from the SEER database, this study systematically compared the long-term prognosis of four treatment modalities—ET, ET + CRT, G, and G + CRT—and performed external validation with an independent multicenter cohort. On this basis, a CSS prediction nomogram composed of age, sex, race, T stage, N stage, RNE, and treatment modality was constructed and validated. The model showed good discrimination and overall calibration after both bootstrap internal validation and validation in an independent external cohort.

In unadjusted analyses, the ET group had higher 5-year CSS than the G group, while the ET + CRT group had the lowest 5-year CSS. After sIPTW adjustment, no statistically significant difference in long-term survival was observed between ET and G in the development cohort, consistent with previous comparative studies [22,23,24,25,26]. This attenuation suggests that treatment selection and differences in baseline tumor characteristics may partly explain the more favorable unadjusted outcomes in the ET group. Patients receiving ET generally had earlier T stage, smaller tumors, and a higher proportion of N0 disease, whereas those undergoing G more often had T1b disease, larger tumors, or a greater risk of occult lymph node metastasis. Japanese, European, and Chinese EGC guidelines all restrict endoscopic resection to intramucosal, very-low-risk, completely resectable lesions, and gastrectomy with lymphadenectomy remains the standard curative approach for beyond-indication cases [27,28,29]. These findings support ET as an organ-preserving option for eligible patients, with treatment selection guided by lymph node metastasis risk and the likelihood of achieving curative endoscopic resection. Notably, in the external cohort, ET was associated with higher 5-year CSS and OS than G, and the sIPTW-adjusted comparison also favored ET. This differed from the development cohort, suggesting that treatment-associated survival estimates may vary with residual confounding or a higher baseline tumor burden. The external findings provide additional real-world evidence of the association between ET and survival, but do not establish its superiority over gastrectomy.

In the overall cohort of this study, patients receiving G + CRT had poorer survival outcomes than those receiving G alone. In both the SEER development and external validation cohorts, 5-year OS and CSS also declined with increasing nodal stage from N1 to N3, highlighting the prognostic importance of nodal burden. The poorer outcomes associated with G + CRT may partly reflect confounding by indication, as postoperative adjuvant therapy is generally considered for patients with lymph node involvement, higher pathological stage, or other high-risk features [27,28]. Previous real-world studies have also shown that postoperative adjuvant therapy for gastric cancer is not randomly assigned, and imbalances in the number of positive lymph nodes, pathological TNM stage, and extent of lymph node dissection commonly exist across treatment groups [30,31].

Notably, when the study population was restricted to node-positive patients, the G + CRT group had lower cancer-specific mortality in both the SEER and external validation cohorts. This finding suggests a potential role for postoperative treatment in selected patients with nodal involvement, although it should be interpreted as an observational association. Previous studies on the value of postoperative adjuvant therapy in EGC have reached inconsistent conclusions. Several studies found no significant difference in CSS among gastrectomy alone, adjuvant chemotherapy, and adjuvant chemoradiotherapy after standardized gastrectomy and lymph node dissection [32,33,34]. Other studies found that adjuvant chemotherapy or chemoradiotherapy was associated with better OS [30,35]. Although current guidelines do not recommend routine postoperative chemoradiotherapy for all EGC patients [36], individualized consideration may be reasonable for positive margins, inadequate dissection, or high locoregional recurrence risk.

This study showed that older age was associated with a higher risk of cancer-specific death, and the increased risk was mainly concentrated in patients over 75 years. Previous SEER studies also showed that the risk of cancer-specific death increases with age, and the age effect is more pronounced in stage I gastric cancer [37]. The CSS HR for male versus female patients was 1.41, but the difference did not reach statistical significance, suggesting a trend toward a higher risk of cancer-specific death in males. The impact of sex on gastric cancer prognosis remains inconclusive, possibly related to lifestyle, hormonal status, histological type, and various other factors [38,39,40]. Regarding race, Black and White patients had similar risks, while patients of other races (mainly Asian) had a lower risk, possibly reflecting a combination of screening patterns, treatment quality, and socioeconomic factors [41,42].

Regarding T stage, T1b disease was associated with a significantly higher risk of death than T1a disease, consistent with the association between greater invasion depth and poorer prognosis [43]. N stage also showed strong risk stratification, with markedly elevated predicted risk in patients with N2 and N3 disease. Previous studies have also shown that N stage is a core factor in EGC prognosis assessment [44,45].

RNE was a key independent prognostic factor in this model: compared with those with no lymph nodes examined, patients with >30 nodes examined had a significantly lower CSS risk, confirming the importance of adequate lymph node assessment for prognosis. This finding is largely consistent with the Korean gastric cancer guidelines, which state that at least 30 lymph nodes should be examined and assessed to determine regional lymph node metastasis [46]. The NCCN guidelines and related studies also indicate that examining more than 15 lymph nodes is required to ensure the accuracy of N staging [3], and patients have better CSS and OS than those with ≤15 nodes examined [47]. Other studies have pointed out that examining ≤ 15 or >30 lymph nodes is associated with poorer CSS and OS [48]. Insufficient RNE may lead to ‘stage migration’—that is, underestimation of the true N stage due to inadequate lymph node retrieval, causing some node-positive patients to be misclassified as N0, thereby affecting prognostic assessment and comparability across studies [49].

Margin status was also associated with gastric cancer-specific outcomes in the external cohort. After multivariable Cox adjustment, non-R0 resection was associated with a higher risk of cancer-specific death than R0 resection, a finding broadly consistent with previous studies [50,51]. However, a study of 267 gastric cancer patients found that patients with positive margins had a significantly shorter median OS than patients who underwent R0 resection, but after multivariable analysis margin status was no longer an independent prognostic factor, possibly because positive margins reflect more advanced and aggressive tumor biology [52]. Several postoperative chemotherapy regimens were used in the external cohort, reflecting the heterogeneity of real-world treatment practice. Previous randomized trials have established the efficacy of S-1 and XELOX as adjuvant therapy after D2 gastrectomy [53,54], and the ARTIST 2 trial further showed that, in node-positive gastric cancer patients, the SOX regimen conferred a disease-free survival benefit over S-1 [55]. Notably, in ARTIST 2, adding radiotherapy to SOX did not yield a further significant improvement in disease-free survival [55]. In our node-positive subgroup, numerical differences in OS were observed among S-1, SOX, XELOX, and other regimens, but these differences were not statistically significant. Current evidence does not support that one oxaliplatin-based adjuvant regimen is clearly superior to another; therefore, our findings should be considered exploratory rather than comparative evidence of regimen efficacy. In daily clinical practice, regimen selection may be influenced by nodal burden, age, physical condition, comorbidities, treatment tolerance, and physician preference, which may lead to confounding by indication.

To our knowledge, this is the first model to simultaneously predict CSS across all four treatment modalities, with good discrimination and calibration. Its strengths are threefold: first, development in a large population-based registry with independent multicenter external validation; second, mitigation of treatment-selection bias via sIPTW supported by systematic subgroup analyses; and third, use of routine variables paired with a web-based calculator for bedside estimation. Clinically, the nomogram supports individualized post-treatment risk stratification and shared decision-making between endoscopic and surgical options and may prompt closer surveillance for high-risk patients complementing rather than replacing guidelines and clinical judgment.

This study has the following limitations. First, the data were derived from the SEER database and retrospectively collected records from the participating centers, which may entail selection bias. Second, SEER’s coding resolution for endoscopic resection is limited and cannot distinguish endoscopic mucosal resection (EMR) from ESD. Because EMR has a lower complete-resection rate than ESD [56] and a higher local recurrence rate [57], grouping both as ‘endoscopic therapy’ may introduce within-group heterogeneity. Moreover, SEER records only first-course treatment, which precludes tracking of salvage gastrectomy after recurrence, and may therefore underestimate the impact of subsequent therapy on long-term survival. Third, the external validation cohort lacks multi-ethnic representation and may not reflect global population characteristics, necessitating further validation in multi-ethnic cohorts including Western populations. Fourth, the sIPTW analysis in this study did not cover all treatment modalities, and only unadjusted survival analyses were performed for the small ET + CRT group. Future studies could uniformly compare the four treatment strategies using a larger ET + CRT sample to further assess the consistency of the findings.

In this study, no statistically significant difference in long-term survival between ET and G was observed after sIPTW adjustment in the development cohort, although ET was associated with better survival outcomes in the external validation cohort. G + CRT was associated with poorer survival in the overall population, whereas among node-positive patients, it was associated with a lower risk of cancer-specific death than G alone in both cohorts. The CSS prediction model demonstrated good predictive performance after internal and external validation and may facilitate individualized prognostic assessment in patients with EGC.

## 5. Conclusions

In this population-based and multicenter study, we compared long-term survival outcomes across different treatment strategies for early gastric cancer and developed an externally validated model for predicting cancer-specific survival. After adjustment for measured confounders, no statistically significant difference in cancer-specific survival was observed between endoscopic therapy and gastrectomy in the SEER cohort, whereas endoscopic therapy was associated with better survival outcomes in the external cohort. Among patients with lymph node metastasis, gastrectomy combined with chemoradiotherapy was associated with lower cancer-specific mortality than gastrectomy alone in both the development and external cohorts, although these observational findings require cautious interpretation. The prognostic model demonstrated good discrimination in an independent multicenter cohort and was implemented as a web-based calculator for individualized survival estimation. Further studies are warranted to assess the comparative effectiveness of treatment strategies and the model’s clinical applicability in broader patient populations.

## Figures and Tables

**Table 1:** Baseline characteristics stratified by therapy.

Variable	Category	Total (*n* = 1766)	Endoscopic Therapy (*n* = 444)	Endoscopic Therapy + CRT (*n* = 44)	Gastrectomy (*n* = 995)	Gastrectomy + CRT (*n* = 283)	*p*
Age, years, *n* (%)	<55	233 (13.2)	25 (5.6)	2 (4.5)	151 (15.2)	55 (19.4)	<0.001
	55–64	367 (20.8)	95 (21.4)	5 (11.4)	189 (19.0)	78 (27.6)	
	65–74	599 (33.9)	139 (31.3)	15 (34.1)	352 (35.4)	93 (32.9)	
	75–84	460 (26.0)	135 (30.4)	19 (43.2)	253 (25.4)	53 (18.7)	
	≥85	107 (6.1)	50 (11.3)	3 (6.8)	50 (5.0)	4 (1.4)	
Sex, *n* (%)	Male	1094 (61.9)	320 (72.1)	34 (77.3)	573 (57.6)	167 (59.0)	<0.001
	Female	672 (38.1)	124 (27.9)	10 (22.7)	422 (42.4)	116 (41.0)	
Race, *n* (%)	White	1199 (67.9)	339 (76.4)	36 (81.8)	643 (64.6)	181 (64.0)	<0.001
	Black	186 (10.5)	36 (8.1)	2 (4.5)	109 (11.0)	39 (13.8)	
	Other	381 (21.6)	69 (15.5)	6 (13.6)	243 (24.4)	63 (22.3)	
Marital status, *n* (%)	Married	1096 (62.1)	282 (63.5)	25 (56.8)	611 (61.4)	178 (62.9)	0.649
	Single	309 (17.5)	67 (15.1)	7 (15.9)	186 (18.7)	49 (17.3)	
	Other	361 (20.4)	95 (21.4)	12 (27.3)	198 (19.9)	56 (19.8)	
Primary site, *n* (%)	Cardia	591 (33.5)	266 (59.9)	38 (86.4)	195 (19.6)	92 (32.5)	<0.001
	Pylorus	58 (3.3)	7 (1.6)	0 (0.0)	39 (3.9)	12 (4.2)	
	Other	1117 (63.3)	171 (38.5)	6 (13.6)	761 (76.5)	179 (63.3)	
Histology, *n* (%)	Adenocarcinoma	1458 (82.6)	422 (95.0)	43 (97.7)	762 (76.6)	231 (81.6)	<0.001
	Signet ring cell carcinoma	231 (13.1)	20 (4.5)	1 (2.3)	174 (17.5)	36 (12.7)	
	Other	77 (4.4)	2 (0.5)	0 (0.0)	59 (5.9)	16 (5.7)	
Tumor size, *n* (%)	≤2 cm	1163 (65.9)	387 (87.2)	25 (56.8)	656 (65.9)	95 (33.6)	<0.001
	2–3 cm	278 (15.7)	30 (6.8)	7 (15.9)	170 (17.1)	71 (25.1)	
	>3 cm	325 (18.4)	27 (6.1)	12 (27.3)	169 (17.0)	117 (41.3)	
T stage, *n* (%)	T1a	836 (47.3)	308 (69.4)	12 (27.3)	447 (44.9)	69 (24.4)	<0.001
	T1b	930 (52.7)	136 (30.6)	32 (72.7)	548 (55.1)	214 (75.6)	
N stage, *n* (%)	N0	1546 (87.5)	443 (99.8)	42 (95.5)	942 (94.7)	119 (42.0)	<0.001
	N1	149 (8.4)	1 (0.2)	2 (4.5)	35 (3.5)	111 (39.2)	
	N2	50 (2.8)	0 (0.0)	0 (0.0)	16 (1.6)	34 (12.0)	
	N3	21 (1.2)	0 (0.0)	0 (0.0)	2 (0.2)	19 (6.7)	
AJCC stage, *n* (%)	I	1656 (93.8)	443 (99.8)	43 (97.7)	969 (97.4)	201 (71.0)	<0.001
	II	102 (5.8)	1 (0.2)	1 (2.3)	23 (2.3)	77 (27.2)	
	III	8 (0.5)	0 (0.0)	0 (0.0)	3 (0.3)	5 (1.8)	
	IV	0 (0.0)	0 (0.0)	0 (0.0)	0 (0.0)	0 (0.0)	
Regional nodes examined, *n* (%)	No examined	554 (31.4)	429 (96.6)	42 (95.5)	72 (7.2)	11 (3.9)	<0.001
	Biopsy only	9 (0.5)	7 (1.6)	1 (2.3)	1 (0.1)	0 (0.0)	
	≤15	458 (25.9)	4 (0.9)	1 (2.3)	367 (36.9)	86 (30.4)	
	16–30	533 (30.2)	3 (0.7)	0 (0.0)	399 (40.1)	131 (46.3)	
	>30	212 (12.0)	1 (0.2)	0 (0.0)	156 (15.7)	55 (19.4)	
Diagnosis-to-treatment time, *n* (%)	≤1 month	713 (40.4)	279 (62.8)	20 (45.5)	332 (33.4)	82 (29.0)	<0.001
	1–3 months	841 (47.6)	128 (28.8)	19 (43.2)	513 (51.6)	181 (64.0)	
	3–6 months	175 (9.9)	31 (7.0)	3 (6.8)	124 (12.5)	17 (6.0)	
	>6 months	37 (2.1)	6 (1.4)	2 (4.5)	26 (2.6)	3 (1.1)	

AJCC, American Joint Committee on Cancer; CRT, chemoradiotherapy; N, regional lymph node category; T, primary tumor category. Values are shown as *n* (%). *p* values were calculated using chi-square tests.

## Data Availability

The SEER data analyzed in this study are publicly available from the Surveillance, Epidemiology, and End Results Program of the National Cancer Institute. The raw data supporting the findings from the multicenter external validation cohort are available from the corresponding authors upon reasonable request.

## Supplementary Materials

The following supporting information can be downloaded at: https://www.mdpi.com/article/10.3390/cancers18213488/s1, Figure S1: Unadjusted Kaplan–Meier curves comparing different treatment strategies for cancer-specific survival in early gastric cancer; Figure S2: Unadjusted Kaplan–Meier curves comparing different treatment strategies for overall survival in early gastric cancer; Figure S3: Unadjusted Kaplan–Meier curves comparing different treatment strategies for cancer-specific survival in the external validation cohort; Figure S4: Unadjusted Kaplan–Meier curves comparing different treatment strategies for overall survival in the external validation cohort; Figure S5: Stabilized inverse probability of treatment weighting-adjusted Kaplan–Meier curves comparing cancer-specific and overall survival between treatment groups in the external validation cohort; Figure S6: Multivariable Cox proportional hazards analyses for cancer-specific and overall survival in the external validation cohort; Figure S7: Kaplan–Meier curves comparing overall and cancer-specific survival according to N stage in the SEER development cohort; Figure S8: Kaplan–Meier curves comparing overall and cancer-specific survival according to N stage in the external validation cohort; Figure S9: Kaplan–Meier curves comparing gastrectomy plus chemoradiotherapy with gastrectomy alone for cancer-specific and overall survival in patients with lymph node-positive early gastric cancer; Figure S10: Variable selection using LASSO regression and Boruta analysis and the final multivariable Cox regression model for predicting cancer-specific survival in early gastric cancer; Figure S11: Decision curve analysis of the nomogram for predicting cancer-specific survival in the internal and external validation cohorts; Table S1: Baseline characteristics of the external validation cohort stratified by therapy; Table S2: Five-year cancer-specific and overall survival rates stratified by treatment strategy in the SEER development cohort; Table S3: Covariate balance before and after stabilized inverse probability of treatment weighting for endoscopic therapy versus gastrectomy in the SEER development cohort; Table S4: Stabilized inverse probability of treatment weighting diagnostics and weighted Cox regression results in the SEER development cohort; Table S5: Covariate balance before and after stabilized inverse probability of treatment weighting for gastrectomy versus gastrectomy plus chemoradiotherapy in the SEER development cohort; Table S6: Five-year cancer-specific and overall survival rates stratified by treatment strategy in the external validation cohort; Table S7: Covariate balance before and after stabilized inverse probability of treatment weighting for endoscopic therapy versus gastrectomy in the external validation cohort; Table S8: Covariate balance before and after stabilized inverse probability of treatment weighting for gastrectomy versus gastrectomy plus chemoradiotherapy in the external validation cohort; Table S9: Stabilized inverse probability of treatment weighting diagnostics and weighted Cox regression results in the external validation cohort; Table S10: Distribution of chemotherapy regimens among patients receiving chemoradiotherapy; Table S11: Exploratory survival outcomes according to chemotherapy regimen among patients receiving gastrectomy plus chemoradiotherapy; Table S12: Univariable and multivariable Cox proportional hazards analyses for cancer-specific survival in the external cohort; Table S13: Univariable and multivariable Cox proportional hazards analyses for overall survival in the external cohort; Table S14: Baseline characteristics of patients with lymph node-positive early gastric cancer in the SEER and external validation cohorts; Table S15: Baseline characteristics of patients with lymph node-positive early gastric cancer stratified by N stage in the SEER cohort; Table S16: Baseline characteristics of patients with lymph node-positive early gastric cancer stratified by N stage in the external validation cohort; Table S17: Five-year overall and cancer-specific survival rates according to N stage; Table S18: Cox regression and log-rank analyses according to N stage; Table S19: Chemotherapy regimen distribution according to nodal stage among node-positive patients receiving gastrectomy plus chemoradiotherapy in the external validation cohort; Table S20: Survival outcomes according to chemotherapy regimen among node-positive patients receiving gastrectomy plus chemoradiotherapy in the external validation cohort; Table S21: Univariable Cox regression analysis for cancer-specific survival; Table S22: Comparison of variable selection methods and the final prediction model; Table S23: Multivariable Cox regression analysis for cancer-specific survival.

## Abbreviations

The following abbreviations are used in this manuscript:

AIC	Akaike information criterion
AJCC	American Joint Committee on Cancer
AUC	area under the curve
BSR	best-subset regression
CI	confidence interval
C-index	concordance index
CRT	chemoradiotherapy
CSS	cancer-specific survival
DCA	decision curve analysis
DTTT	diagnosis-to-treatment time
EGC	early gastric cancer
EMR	endoscopic mucosal resection
ESD	endoscopic submucosal dissection
ET	endoscopic therapy
ET + CRT	endoscopic therapy plus chemoradiotherapy
G	gastrectomy
G + CRT	gastrectomy plus chemoradiotherapy
HR	hazard ratio
ICD-O-3	International Classification of Diseases for Oncology, Third Edition
LASSO	least absolute shrinkage and selection operator
NCI	National Cancer Institute
NCCN	National Comprehensive Cancer Network
OS	overall survival
RNE	number of regional lymph nodes examined
ROC	receiver operating characteristic
SEER	Surveillance, Epidemiology, and End Results
SMD	standardized mean difference
sIPTW	stabilized inverse probability of treatment weighting
TRIPOD + AI	Transparent Reporting of a multivariable prediction model for Individual Prognosis Or Diagnosis–Artificial Intelligence
