# Supplementary material for: Treatment Effects of Different Therapeutic Strategies and Development of a Prognostic Prediction Model for Early Gastric Cancer: A Population-Based and Multicenter Study
Source: Cancers (Basel). 2026 Oct 29;18(21):3488. doi: 10.3390/cancers18213488 (PMC13761578; doi:10.3390/cancers18213488)
Supplement: Supplementary file 1 [file cancers-18-03488-s001.zip › cancers-4618820-supplementary.pdf]

Table S1. Baseline Characteristics of the External Validation Cohort Stratified by Therapy

Variable	Category	Total (n=1333)	ET (n=517)	ET+CRT (n=22)	G (n=613)	G+CRT (n=181)	P
Age, years	<55	250 (18.8)	64 (12.4)	3 (13.6)	129 (21.0)	54 (29.8)	<0.001
	55-64	426 (32.0)	184 (35.6)	9 (40.9)	179 (29.2)	54 (29.8)	
	65-74	482 (36.2)	197 (38.1)	8 (36.4)	218 (35.6)	59 (32.6)	
	75-84	154 (11.6)	62 (12.0)	2 (9.1)	76 (12.4)	14 (7.7)	
	≥85	21 (1.6)	10 (1.9)	0 (0.0)	11 (1.8)	0 (0.0)	
Sex	Male	911 (68.3)	376 (72.7)	16 (72.7)	405 (66.1)	114 (63.0)	0.034
	Female	422 (31.7)	141 (27.3)	6 (27.3)	208 (33.9)	67 (37.0)	
Race	White	17 (1.3)	11 (2.1)	0 (0.0)	5 (0.8)	1 (0.6)	0.168
	Other	1316 (98.7)	506 (97.9)	22 (100.0)	608 (99.2)	180 (99.4)	
	Black	0(0.0)	0(0.0)	0(0.0)	0(0.0)	0(0.0)	
Marital status	Married	1299 (97.4)	516 (99.8)	22 (100.0)	597 (97.4)	164 (90.6)	<0.001
	Single	19 (1.4)	1 (0.2)	0 (0.0)	11 (1.8)	7 (3.9)	
	Other	15 (1.1)	0 (0.0)	0 (0.0)	5 (0.8)	10 (5.5)	
Primary site	Cardia	194 (14.6)	52 (10.1)	5 (22.7)	105 (17.1)	32 (17.7)	<0.001
	Pylorus	18 (1.4)	1 (0.2)	0 (0.0)	14 (2.3)	3 (1.7)	
	Other	1121 (84.1)	464 (89.7)	17 (77.3)	494 (80.6)	146 (80.7)	
Histology	Adenocarcinoma	1155 (86.6)	508 (98.3)	14 (63.6)	485 (79.1)	148 (81.8)	<0.001
	Signet ring cell carcinoma	150 (11.3)	9 (1.7)	8 (36.4)	108 (17.6)	25 (13.8)	
	Other	28 (2.1)	0 (0.0)	0 (0.0)	20 (3.3)	8 (4.4)	
Tumor size	≤2cm	883 (66.2)	391 (75.6)	6 (27.3)	395 (64.4)	91 (50.3)	<0.001
	2-3cm	259 (19.4)	66 (12.8)	5 (22.7)	141 (23.0)	47 (26.0)	
	>3cm	191 (14.3)	60 (11.6)	11 (50.0)	77 (12.6)	43 (23.8)	
T stage	T1a	810 (60.8)	458 (88.6)	2 (9.1)	321 (52.4)	29 (16.0)	<0.001
	T1b	523 (39.2)	59 (11.4)	20 (90.9)	292 (47.6)	152 (84.0)	
N stage	N0	1189 (89.2)	517 (100.0)	13 (59.1)	586 (95.6)	73 (40.3)	<0.001
	N1	84 (6.3)	0 (0.0)	6 (27.3)	16 (2.6)	62 (34.3)	
	N2	43 (3.2)	0 (0.0)	2 (9.1)	7 (1.1)	34 (18.8)	
	N3	17 (1.3)	0 (0.0)	1 (4.5)	4 (0.7)	12 (6.6)	
AJCC stage	I	1269 (95.2)	517 (100.0)	19 (86.4)	602 (98.2)	131 (72.4)	<0.001

Variable	Category	Total (*n*=1333)	ET (*n*=517)	ET+CRT (*n*=22)	G (n=613)	G+CRT (n=181)	*P*
	II	59 (4.4)	0 (0.0)	3 (13.6)	10 (1.6)	46 (25.4)	
	III	5 (0.4)	0 (0.0)	0 (0.0)	1 (0.2)	4 (2.2)	
Regional nodes examined	No examined	527 (39.5)	496 (95.9)	17 (77.3)	12 (2.0)	2 (1.1)	<0.001
	≤15	83 (6.2)	1 (0.2)	0 (0.0)	69 (11.3)	13 (7.2)	
	16-30	203 (15.2)	2 (0.4)	0 (0.0)	150 (24.5)	51 (28.2)	
	>30	520 (39.0)	18 (3.5)	5 (22.7)	382 (62.3)	115 (63.5)	
Diagnosis-to-treatment time	≤1 month	822 (61.7)	330 (63.8)	17 (77.3)	371 (60.5)	104 (57.5)	0.480
	1-3 months	405 (30.4)	143 (27.7)	5 (22.7)	193 (31.5)	64 (35.4)	
	3-6 months	66 (5.0)	25 (4.8)	0 (0.0)	33 (5.4)	8 (4.4)	
	>6 months	40 (3.0)	19 (3.7)	0 (0.0)	16 (2.6)	5 (2.8)	
Resection status	R0 resection	1306 (98.0)	502 (97.1)	17 (77.3)	609 (99.3)	178 (98.3)	<0.001
	Non-R0 resection	27 (2.0)	15 (2.9)	5 (22.7)	4 (0.7)	3 (1.7)	

AJCC, American Joint Committee on Cancer; ET, endoscopic therapy; ET+CRT, endoscopic therapy plus chemoradiotherapy; G, gastrectomy; G+CRT, gastrectomy plus chemoradiotherapy.

Table S2. Five-year CSS and OS rates stratified by therapy method

Group	n	CSS events	5-year CSS, % (95%CI)	OS events	5-year OS, % (95%CI)
ET	444	18	94.0 (90.4-96.3)	85	72.0 (65.2-77.7)
ET + CRT	44	11	61.0 (36.6-78.5)	17	42.6 (21.5-62.4)
G	995	72	90.7 (88.0-92.8)	158	76.5 (72.4-80.1)
G + CRT	283	32	83.3 (75.0-89.0)	54	69.6 (59.0-77.9)

CSS, cancer-specific survival; OS, overall survival; CRT, chemoradiotherapy; CI, confidence interval; ET, endoscopic therapy; ET+CRT, endoscopic therapy plus chemoradiotherapy; G, gastrectomy; G+CRT, gastrectomy plus chemoradiotherapy. Five-year survival rates were estimated by the Kaplan-Meier method at 60 months.

Table S3. Covariate balance before and after sIPTW: Endoscopic therapy vs Gastrectomy

Variable	Category	Unadjusted ET	Unadjusted G	*P*	After sIPTW ET	After sIPTW G	*P*	SMD before	SMD after
**Age (years)**				<0.001			0.950	0.317	0.031
	<55	25 (5.6)	151 (15.2)		48.5 (11.1)	119.4 (12.1)			
	55-64	95 (21.4)	189 (19.0)		89.2 (20.4)	199.2 (20.2)			
	65-74	139 (31.3)	352 (35.4)		159.1 (36.4)	349.0 (35.4)			
	75-84	135 (30.4)	253 (25.4)		108.1 (24.8)	254.0 (25.8)			
	≥85	50 (11.3)	50 (5.0)		31.7 (7.3)	64.7 (6.6)			
**Sex**				<0.001			0.260	0.307	0.065
	Female	124 (27.9)	422 (42.4)		148.9 (34.1)	367.0 (37.2)			
	Male	320 (72.1)	573 (57.6)		287.7 (65.9)	619.2 (62.8)			
**Race**				<0.001			0.881	0.259	0.029
	White	339 (76.4)	643 (64.6)		289.0 (66.2)	666.1 (67.5)			
	Black	36 (8.1)	109 (11.0)		46.4 (10.6)	100.4 (10.2)			
	Other	69 (15.5)	243 (24.4)		101.3 (23.2)	219.7 (22.3)			
**Marital status**				0.244			0.223	0.096	0.084
	Married	282 (63.5)	611 (61.4)		272.4 (62.4)	621.0 (63.0)			
	Single	67 (15.1)	186 (18.7)		91.0 (20.8)	173.0 (17.5)			
	Other	95 (21.4)	198 (19.9)		73.2 (16.8)	192.2 (19.5)			
**Primary site**				<0.001			0.456	0.904	0.064
	Cardia	266 (59.9)	195 (19.6)		140.8 (32.3)	308.6 (31.3)			
	Pylorus	7 (1.6)	39 (3.9)		19.2 (4.4)	31.3 (3.2)			
	Other	171 (38.5)	761 (76.5)		276.6 (63.4)	646.3 (65.5)			
**Histology**				<0.001			0.018	0.549	0.150
	Adenocarcinoma	422 (95.0)	762 (76.6)		380.9 (87.2)	811.1 (82.2)			
	Signet ring cell carcinoma	20 (4.5)	174 (17.5)		48.2 (11.1)	133.1 (13.5)			
	Other	2 (0.5)	59 (5.9)		7.4 (1.7)	41.9 (4.3)			
**Tumor size**				<0.001			0.070	0.518	0.131
	≤2 cm	387 (87.2)	656 (65.9)		325.9 (74.6)	714.6 (72.5)			
	2-3 cm	30 (6.8)	170 (17.1)		41.9 (9.6)	136.0 (13.8)			
	>3 cm	27 (6.1)	169 (17.0)		68.8 (15.8)	135.6 (13.7)			
**T stage**				<0.001			0.277	0.510	0.063
	T1a	308 (69.4)	447 (44.9)		212.4 (48.7)	510.7 (51.8)			
	T1b	136 (30.6)	548 (55.1)		224.2 (51.3)	475.5 (48.2)			
**Diagnosis-to-treatment time**				<0.001			0.111	0.617	0.119

Variable	Category	Unadjusted ET	Unadjusted G	*P*	After sIPTW ET	After sIPTW G	*P*	SMD before	SMD after
	≤1 month	279 (62.8)	332 (33.4)		197.8 (45.3)	413.1 (41.9)			
	1-3 months	128 (28.8)	513 (51.6)		169.3 (38.8)	440.1 (44.6)			
	3-6 months	31 (7.0)	124 (12.5)		52.9 (12.1)	110.3 (11.2)			
	>6 months	6 (1.4)	26 (2.6)		16.7 (3.8)	22.7 (2.3)			

sIPTW, stabilized inverse probability of treatment weighting; SMD, standardized mean difference; ET, endoscopic therapy; G, gastrectomy.

Table S4. sIPTW weight diagnostics and weighted Cox results

Comparison	*n*	ESS	Maximum weight	Maximum SMD after sIPTW	Endpoint	HR (95% CI)	*P*
ET vs G	1439	824.5	10.83	0.150	CSS	1.07 (95% CI 0.51-2.27)	0.856
ET vs G	1439	824.5	10.83	0.150	OS	0.77 (95% CI 0.54-1.12)	0.175
G vs G+CRT	1278	1026.6	6.53	0.123	CSS	1.82 (95% CI 1.07-3.10)	0.028
G vs G+CRT	1278	1026.6	6.53	0.123	OS	1.47 (95% CI 1.01-2.15)	0.043

CSS, cancer-specific survival; OS, overall survival; sIPTW, stabilized inverse probability of treatment weighting; ESS, effective sample size; SMD, standardized mean difference; HR, hazard ratio; CI, confidence interval; ET, endoscopic therapy; G, gastrectomy; G+CRT, gastrectomy plus chemoradiotherapy.

Table S5. Covariate balance before and after sIPTW: Gastrectomy vs Gastrectomy + CRT

Variable	Category	Unadjusted G	Unadjusted G+CRT	*P*	After sIPTW G	After sIPTW G+CRT	*P*	SMD before	SMD after
**Age (years)**				<0.001			0.553	0.206	0.109
	<55	151 (15.2)	55 (19.4)		157.7 (15.8)	34.0 (12.0)			
	55-64	189 (19.0)	78 (27.6)		212.5 (21.3)	59.8 (21.2)			
	65-74	352 (35.4)	93 (32.9)		346.9 (34.8)	102.6 (36.3)			
	75-84	253 (25.4)	53 (18.7)		238.9 (23.9)	70.8 (25.1)			
	≥85	50 (5.0)	4 (1.4)		42.1 (4.2)	15.2 (5.4)			
**Sex**				0.669			0.701	0.029	0.026
	Female	422 (42.4)	116 (41.0)		418.1 (41.9)	121.9 (43.2)			
	Male	573 (57.6)	167 (59.0)		580.0 (58.1)	160.5 (56.8)			
**Race**				0.374			0.395	0.086	0.092
	White	643 (64.6)	181 (64.0)		644.6 (64.6)	194.6 (68.9)			
	Black	109 (11.0)	39 (13.8)		115.3 (11.6)	29.7 (10.5)			
	Other	243 (24.4)	63 (22.3)		238.1 (23.9)	58.1 (20.6)			
**Marital status**				0.857			0.666	0.036	0.058
	Married	611 (61.4)	178 (62.9)		620.7 (62.2)	183.5 (65.0)			
	Single	186 (18.7)	49 (17.3)		181.0 (18.1)	45.8 (16.2)			
	Other	198 (19.9)	56 (19.8)		196.3 (19.7)	53.1 (18.8)			
**Primary site**				<0.001			0.988	0.297	0.010
	Cardia	195 (19.6)	92 (32.5)		225.9 (22.6)	65.1 (23.1)			
	Pylorus	39 (3.9)	12 (4.2)		39.7 (4.0)	11.0 (3.9)			
	Other	761 (76.5)	179 (63.3)		732.4 (73.4)	206.3 (73.0)			
**Histology**				0.150			0.840	0.133	0.038
	Adenocarcinoma	762 (76.6)	231 (81.6)		774.4 (77.6)	219.7 (77.8)			
	Signet ring cell carcinoma	174 (17.5)	36 (12.7)		165.6 (16.6)	48.7 (17.3)			
	Other	59 (5.9)	16 (5.7)		58.1 (5.8)	14.0 (5.0)			
**Tumor size**				<0.001			0.947	0.684	0.022
	≤2 cm	656 (65.9)	95 (33.6)		582.8 (58.4)	163.1 (57.7)			
	2-3 cm	170 (17.1)	71 (25.1)		188.9 (18.9)	55.9 (19.8)			
	>3 cm	169 (17.0)	117 (41.3)		226.4 (22.7)	63.5 (22.5)			
**T stage**				<0.001			0.212	0.442	0.085
	T1a	447 (44.9)	69 (24.4)		398.4 (39.9)	101.2 (35.8)			
	T1b	548 (55.1)	214 (75.6)		599.7 (60.1)	181.3 (64.2)			
**Diagnosis-to-treatment time**				<0.001			0.162	0.253	0.123

Variable	Category	Unadjusted G	Unadjusted G+CRT	*P*	After sIPTW G	After sIPTW G+CRT	*P*	SMD before	SMD after
	≤1 month	332 (33.4)	82 (29.0)		317.5 (31.8)	74.1 (26.2)			
	1-3 months	513 (51.6)	181 (64.0)		547.1 (54.8)	165.4 (58.6)			
	3-6 months	124 (12.5)	17 (6.0)		110.3 (11.1)	31.7 (11.2)			
	>6 months	26 (2.6)	3 (1.1)		23.1 (2.3)	11.3 (4.0)			

sIPTW, stabilized inverse probability of treatment weighting; SMD, standardized mean difference; G, gastrectomy; G+CRT, gastrectomy plus chemoradiotherapy.

Table S6. Five-year CSS and OS rates of the External Validation Cohort stratified by therapy

Therapy	n	CSS events	5-year CSS, % (95%CI)	OS events	5-year OS, % (95%CI)
ET	517	6	98.8 (97.4-99.5)	24	95.4 (93.1-96.9)
ET+CRT	22	7	72.7 (49.1-86.7)	9	68.2 (44.6-83.4)
G	613	21	96.7 (94.9-97.8)	53	91.7 (89.2-93.6)
G+CRT	181	14	92.1 (87.0-95.2)	26	85.6 (79.6-90.0)

CSS, cancer-specific survival; OS, overall survival, CI, confidence interval; ET, endoscopic therapy; ET+CRT, endoscopic therapy plus chemoradiotherapy; G, gastrectomy; G+CRT, gastrectomy plus chemoradiotherapy; Five-year survival rates were estimated by the Kaplan-Meier method at 60 months.

Table S7. Covariate balance before and after sIPTW: Endoscopic therapy vs Gastrectomy

Variable	Category	Unadjusted ET	Unadjusted G	*P*	After sIPTW ET	After sIPTW G	*P*	SMD before	SMD after
**Age (years)**				0.002			0.210	0.234	0.140
	<55	64 (12.4)	129 (21.0)		79.2 (15.3)	103.9 (16.3)			
	55-64	184 (35.6)	179 (29.2)		193.0 (37.2)	195.2 (30.6)			
	65-74	197 (38.1)	218 (35.6)		184.2 (35.5)	257.6 (40.4)			
	75-84	62 (12.0)	76 (12.4)		53.0 (10.2)	69.9 (11.0)			
	≥85	10 (1.9)	11 (1.8)		9.7 (1.9)	11.5 (1.8)			
**Sex**				0.016			0.027	0.145	0.132
	Female	141 (27.3)	208 (33.9)		140.3 (27.0)	210.8 (33.0)			
	Male	376 (72.7)	405 (66.1)		378.9 (73.0)	427.3 (67.0)			
**Race**				0.063			0.002	0.109	0.190
	White	11 (2.1)	5 (0.8)		11.5 (2.2)	38.1 (6.0)			
	Other	506 (97.9)	608 (99.2)		507.6 (97.8)	600.1 (94.0)			
**Marital status**				0.004			0.283	0.207	0.092
	Married	516 (99.8)	597 (97.4)		515.4 (99.3)	628.9 (98.5)			
	Single	1 (0.2)	11 (1.8)		3.8 (0.7)	6.6 (1.0)			
	Other	0 (0.0)	5 (0.8)		0.0 (0.0)	2.7 (0.4)			
**Primary site**				<0.001			<0.001	0.260	0.210
	Cardia	52 (10.1)	105 (17.1)		111.9 (21.6)	86.7 (13.6)			
	Pylorus	1 (0.2)	14 (2.3)		2.8 (0.5)	8.1 (1.3)			
	Other	464 (89.7)	494 (80.6)		404.4 (77.9)	543.3 (85.1)			
**Histology**				<0.001			0.001	0.634	0.186
	Adenocarcinoma	508 (98.3)	485 (79.1)		445.2 (85.8)	563.9 (88.4)			
	Signet ring cell carcinoma	9 (1.7)	108 (17.6)		73.9 (14.2)	63.5 (9.9)			
	Other	0 (0.0)	20 (3.3)		0.0 (0.0)	10.9 (1.7)			
**Tumor size**				<0.001			0.085	0.270	0.124
	≤2 cm	391 (75.6)	395 (64.4)		372.5 (71.8)	426.3 (66.8)			
	2-3 cm	66 (12.8)	141 (23.0)		94.1 (18.1)	121.5 (19.0)			
	>3 cm	60 (11.6)	77 (12.6)		52.5 (10.1)	90.3 (14.2)			
**T stage**				<0.001			0.235	0.865	0.070
	T1a	458 (88.6)	321 (52.4)		347.6 (67.0)	448.1 (70.2)			

Variable	Category	Unadjusted ET	Unadjusted G	*P*	After sIPTW ET	After sIPTW G	*P*	SMD before	SMD after
	T1b	59 (11.4)	292 (47.6)		171.5 (33.0)	190.1 (29.8)			
**Diagnosis-to-treatment time**				0.382			0.523	0.084	0.083
	≤1 month	330 (63.8)	371 (60.5)		344.5 (66.4)	406.9 (63.8)			
	1-3 months	143 (27.7)	193 (31.5)		127.6 (24.6)	180.3 (28.2)			
	3-6 months	25 (4.8)	33 (5.4)		33.4 (6.4)	34.5 (5.4)			
	>6 months	19 (3.7)	16 (2.6)		13.7 (2.6)	16.4 (2.6)			

sIPTW, stabilized inverse probability of treatment weighting; SMD, standardized mean difference; ET, endoscopic therapy; G, gastrectomy.

Table S8. Covariate balance before and after sIPTW: Gastrectomy vs Gastrectomy + CRT

Variable	Category	Unadjusted G	Unadjusted G+CRT	*P*	After sIPTW G	After sIPTW G+CRT	*P*	SMD before	SMD after
**Age (years)**				0.027			0.642	0.203	0.167
	<55	129 (21.0)	54 (29.8)		154.7 (24.9)	41.7 (24.2)			
	55-64	179 (29.2)	54 (29.8)		178.8 (28.8)	52.0 (30.1)			
	65-74	218 (35.6)	59 (32.6)		209.7 (33.8)	59.9 (34.7)			
	75-84	76 (12.4)	14 (7.7)		69.4 (11.2)	18.9 (11.0)			
	≥85	11 (1.8)	0 (0.0)		8.5 (1.4)	0.0 (0.0)			
**Sex**				0.443			0.126	0.065	0.131
	Female	208 (33.9)	67 (37.0)		216.4 (34.8)	71.1 (41.2)			
	Male	405 (66.1)	114 (63.0)		404.7 (65.2)	101.5 (58.8)			
**Race**				0.223			0.299	0.128	0.112
	White	5 (0.8)	0 (0.0)		3.9 (0.6)	0.0 (0.0)			
	Other	608 (99.2)	181 (100.0)		617.3 (99.4)	172.6 (100.0)			
**Marital status**				<0.001			0.820	0.289	0.055
	Married	597 (97.4)	164 (90.6)		587.0 (94.5)	165.2 (95.7)			
	Single	11 (1.8)	7 (3.9)		21.1 (3.4)	4.4 (2.6)			
	Other	5 (0.8)	10 (5.5)		13.0 (2.1)	3.0 (1.7)			
**Primary site**				0.869			0.862	0.045	0.047
	Cardia	105 (17.1)	32 (17.7)		103.7 (16.7)	25.8 (15.0)			
	Pylorus	14 (2.3)	3 (1.7)		13.3 (2.1)	3.7 (2.2)			
	Other	494 (80.6)	146 (80.7)		504.1 (81.2)	143.1 (82.9)			
**Histology**				0.394			0.057	0.105	0.195
	Adenocarcinoma	485 (79.1)	148 (81.8)		496.1 (79.9)	125.9 (72.9)			
	Signet ring cell carcinoma	108 (17.6)	25 (13.8)		104.6 (16.8)	42.7 (24.7)			
	Other	20 (3.3)	8 (4.4)		20.4 (3.3)	4.1 (2.4)			
**Tumor size**				<0.001			0.535	0.294	0.084
	≤2 cm	395 (64.4)	91 (50.3)		378.3 (60.9)	104.2 (60.4)			
	2-3 cm	141 (23.0)	47 (26.0)		144.8 (23.3)	35.6 (20.6)			
	>3 cm	77 (12.6)	43 (23.8)		98.1 (15.8)	32.7 (19.0)			
**T stage**				<0.001			0.948	0.829	0.006
	T1a	321 (52.4)	29 (16.0)		271.3 (43.7)	75.9 (44.0)			
	T1b	292 (47.6)	152 (84.0)		349.8 (56.3)	96.7 (56.0)			
**Diagnosis-to-treatment time**				0.770			0.306	0.082	0.125
	≤1 month	371 (60.5)	104 (57.5)		365.2 (58.8)	93.5 (54.2)			

Variable	Category	Unadjusted G	Unadjusted G+CRT	*P*	After sIPTW G	After sIPTW G+CRT	*P*	SMD before	SMD after
	1-3 months	193 (31.5)	64 (35.4)		207.1 (33.3)	57.8 (33.5)			
	3-6 months	33 (5.4)	8 (4.4)		32.0 (5.2)	14.3 (8.3)			
	>6 months	16 (2.6)	5 (2.8)		16.8 (2.7)	7.1 (4.1)			

sIPTW, stabilized inverse probability of treatment weighting; SMD, standardized mean difference; G, gastrectomy; G+CRT, gastrectomy plus chemoradiotherapy.

Table S9. sIPTW weight diagnostics and weighted Cox results

Comparison	*n*	ESS	Maximum weight	Maximum SMD after sIPTW	Endpoint	HR (95% CI)	*P*	1%-99% trimmed HR (95% CI)	Trimmed *P*
ET vs G	1130	346.3	41.33	0.210	CSS	2.29 (95% CI 0.81-6.46)	0.117	2.14 (95% CI 0.77-5.93)	0.144
ET vs G	1130	346.3	41.33	0.210	OS	2.08 (95% CI 1.18-3.68)	0.012	1.94 (95% CI 1.13-3.33)	0.016
G vs G+CRT	794	586.3	7.12	0.195	CSS	1.85 (95% CI 0.89-3.85)	0.099	1.92 (95% CI 0.93-3.96)	0.079
G vs G+CRT	794	586.3	7.12	0.195	OS	1.50 (95% CI 0.85-2.64)	0.158	1.56 (95% CI 0.89-2.71)	0.118

CSS, cancer-specific survival; OS, overall survival; sIPTW, stabilized inverse probability of treatment weighting; ESS, effective sample size; SMD, standardized mean difference; HR, hazard ratio; CI, confidence interval; ET, endoscopic therapy; G, gastrectomy; G+CRT, gastrectomy plus chemoradiotherapy.

Table S10. Distribution of chemotherapy regimens among patients receiving chemoradiotherapy

Regimen	Overall CRT (n = 203)	ET+CRT (n = 22)	G+CRT (n = 181)	OS deaths, n (%)	CSS deaths, n (%)
S-1	82 (40.4%)	9 (40.9%)	73 (40.3%)	9 (11.0%)	5 (6.1%)
SOX	53 (26.1%)	8 (36.4%)	45 (24.9%)	8 (15.1%)	6 (11.3%)
XELOX	35 (17.2%)	5 (22.7%)	30 (16.6%)	12 (34.3%)	6 (17.1%)
TS	17 (8.4%)	0 (0.0%)	17 (9.4%)	3 (17.6%)	2 (11.8%)
DOS	8 (3.9%)	0 (0.0%)	8 (4.4%)	1 (12.5%)	1 (12.5%)
FOLFOX	3 (1.5%)	0 (0.0%)	3 (1.7%)	1 (33.3%)	1 (33.3%)
DS	3 (1.5%)	0 (0.0%)	3 (1.7%)	0 (0.0%)	0 (0.0%)
CAP	2 (1.0%)	0 (0.0%)	2 (1.1%)	1 (50.0%)	0 (0.0%)

CSS, cancer-specific survival; OS, overall survival; S-1, an oral fluoropyrimidine derivative (tegafur/gimeracil/oteracil); SOX, S-1 plus oxaliplatin; XELOX, capecitabine plus oxaliplatin; TS, liposomal paclitaxel plus S-1; DOS, docetaxel, oxaliplatin, and S-1; FOLFOX, folinic acid, fluorouracil, and oxaliplatin; DS, docetaxel plus S-1; CAP, capecitabine; ET+CRT, endoscopic therapy plus chemoradiotherapy; G+CRT, gastrectomy plus chemoradiotherapy.

Table S11. Exploratory survival outcomes according to chemotherapy regimen among all patients receiving gastrectomy plus chemoradiotherapy

Regimen	*n*	OS deaths	1-year OS (95% CI)	3-year OS (95% CI)	5-year OS (95% CI)	CSS deaths	1-year CSS (95% CI)	3-year CSS (95% CI)	5-year CSS (95% CI)
S-1	73	7	94.5% (86.1-97.9)	93.2% (84.3-97.1)	90.4% (80.9-95.3)	4	95.9% (87.8-98.7)	95.9% (87.8-98.7)	94.5% (85.9-97.9)
SOX	45	5	97.8% (85.3-99.7)	93.3% (80.7-97.8)	88.9% (75.3-95.2)	3	100.0% (100.0-100.0)	95.5% (83.0-98.8)	93.2% (80.3-97.7)
XELOX	30	8	93.3% (75.9-98.3)	80.0% (60.8-90.5)	73.3% (53.7-85.7)	3	96.7% (78.6-99.5)	89.6% (71.2-96.5)	89.6% (71.2-96.5)
Other	33	6	100.0% (100.0-100.0)	84.8% (67.4-93.4)	81.8% (63.9-91.4)	4	100.0% (100.0-100.0)	87.9% (70.9-95.3)	87.9% (70.9-95.3)

CSS, cancer-specific survival; OS, overall survival; CI, confidence interval; S-1, an oral fluoropyrimidine derivative (tegafur/gimeracil/oteracil); SOX, S-1 plus oxaliplatin; XELOX, capecitabine plus oxaliplatin; Other: TS, DOS, FOLFOX, DS, and CAP.

Table S12. Univariable and Multivariable Cox Proportional Hazards Analyses for Cancer-Specific Survival in the External Cohort

Variable	Category	Univariable analysis			Multivariable analysis		
		HR (95% CI)	*P*	Overall *P*	HR (95% CI)	*P*	Overall *P*
**Age (years)**	<55	Ref.		<0.001	Ref.		<0.001
	55-64	0.286 (0.086-0.950)	0.041		0.397 (0.117-1.350)	0.139	
	65-74	1.167 (0.507-2.684)	0.716		1.555 (0.654-3.695)	0.317	
	75-84	3.508 (1.501-8.200)	0.004		8.203 (3.099-21.715)	<0.001	
	≥85	3.105 (0.659-14.623)	0.152		6.090 (1.129-32.854)	0.036	
**Sex**	Female	Ref.		0.276			
	Male	1.423 (0.741-2.736)	0.290				
**Race**	White	Ref.		0.025			
	Other	0.188 (0.058-0.606)	0.005				
**Marital status**	Married	Ref.		0.513			
	Single	1.547 (0.213-11.213)	0.666				
	Other	NE	NE				
**Primary site**	Cardia	Ref.		0.406			
	Pylorus	1.171 (0.150-9.149)	0.880				
	Other	0.627 (0.312-1.261)	0.191				
**Histology**	Adenocarcinoma	Ref.		0.324			
	Signet ring cell carcinoma	0.898 (0.356-2.268)	0.820				
	Other	NE	NE				
**Tumor size**	≤2 cm	Ref.		<0.001	Ref.		0.503
	2-3 cm	2.534 (1.270-5.055)	0.008		1.375 (0.664-2.844)	0.391	
	>3 cm	3.799 (1.930-7.476)	<0.001		0.863 (0.399-1.865)	0.707	
**T stage**	T1a	Ref.		<0.001	Ref.		<0.001
	T1b	11.349 (4.824-26.699)	<0.001		5.530 (2.132-14.343)	<0.001	
**N stage**	N0	Ref.		<0.001	Ref.		<0.001
	N1	7.330 (3.572-15.041)	<0.001		7.020 (2.744-17.960)	<0.001	
	N2	10.854 (4.854-24.272)	<0.001		12.314 (4.877-31.095)	<0.001	
	N3	25.240 (10.266-62.052)	<0.001		44.190 (14.747-132.420)	<0.001	
**AJCC stage**	I	Ref.		<0.001			
	II	9.958 (5.239-18.928)	<0.001				
	III	19.319 (4.634-80.547)	<0.001				
**Regional nodes examined**	No examined nodes	Ref.		0.020			
	≤15	3.293 (1.236-8.776)	0.017				

Variable	Category	Univariable analysis			Multivariable analysis		
		HR (95% CI)	*P*	Overall *P*	HR (95% CI)	*P*	Overall *P*
	16-30	2.970 (1.355-6.510)	0.007				
	>30	1.456 (0.695-3.051)	0.319				
**Diagnosis-to-treatment time**	≤1 month	Ref.		0.696			
	1-3 months	0.952 (0.503-1.802)	0.880				
	3-6 months	0.834 (0.199-3.496)	0.804				
	>6 months	2.122 (0.646-6.966)	0.215				
**Therapy**	ET	Ref.		<0.001	Ref.		0.004
	ET+CRT	30.731 (10.325-91.471)	<0.001		4.819 (1.283-18.099)	0.020	
	G	3.018 (1.218-7.479)	0.017		1.134 (0.398-3.225)	0.814	
	G+CRT	7.039 (2.705-18.322)	<0.001		0.624 (0.175-2.231)	0.468	
**Resection status**	R0 resection	Ref.		0.020	Ref.		0.050
	Non-R0 resection	4.350 (1.563-12.110)	0.005		3.775 (1.138-12.525)	0.030	

HR, hazard ratio; CI, confidence interval; RNE, regional nodes examined; NE, not estimable because no events occurred in that category; AJCC, American Joint Committee on Cancer; ET, endoscopic therapy; ET+CRT, endoscopic therapy plus chemoradiotherapy; G, gastrectomy; G+CRT, gastrectomy plus chemoradiotherapy. Reference categories are indicated as Ref.

Table S13. Univariable and Multivariable Cox Proportional Hazards Analyses for Overall Survival in the External Cohort

Variable	Category	Univariable analysis			Multivariable analysis		
		HR (95% CI)	*P*	Overall *P*	HR (95% CI)	*P*	Overall *P*
**Age (years)**	<55	Ref.		<0.001	Ref.		<0.001
	55-64	0.477 (0.240-0.946)	0.034		0.536 (0.265-1.085)	0.083	
	65-74	1.389 (0.808-2.388)	0.234		1.518 (0.858-2.688)	0.152	
	75-84	2.652 (1.461-4.816)	0.001		3.414 (1.758-6.630)	<0.001	
	≥85	2.804 (0.949-8.286)	0.062		3.705 (1.163-11.798)	0.027	
**Sex**	Female	Ref.		0.147			
	Male	1.357 (0.889-2.071)	0.157				
**Race**	White	Ref.		0.218			
	Other	0.443 (0.141-1.395)	0.164				
**Marital status**	Married	Ref.		0.143			
	Single	2.014 (0.640-6.344)	0.231				
	Other	NE	NE				
**Primary site**	Cardia	Ref.		0.337			
	Pylorus	1.847 (0.547-6.241)	0.323				
	Other	0.804 (0.490-1.318)	0.386				
**Histology**	Adenocarcinoma	Ref.		0.075			
	Signet ring cell carcinoma	0.924 (0.508-1.681)	0.795				
	Other	NE	NE				
**Tumor size**	≤2 cm	Ref.		0.023	Ref.		0.593
	2-3 cm	1.180 (0.727-1.915)	0.504		0.785 (0.474-1.302)	0.349	
	>3 cm	1.961 (1.243-3.094)	0.004		0.840 (0.503-1.402)	0.504	
**T stage**	T1a	Ref.		<0.001	Ref.		0.003
	T1b	3.172 (2.145-4.691)	<0.001		1.990 (1.245-3.181)	0.004	
**N stage**	N0	Ref.		<0.001	Ref.		<0.001
	N1	3.910 (2.363-6.470)	<0.001		3.499 (1.789-6.845)	<0.001	
	N2	4.638 (2.463-8.734)	<0.001		4.863 (2.308-10.244)	<0.001	
	N3	9.131 (4.206-19.822)	<0.001		13.135 (5.408-31.907)	<0.001	
**AJCC stage**	I	Ref.		<0.001			
	II	4.673 (2.786-7.838)	<0.001				
	III	7.024 (1.730-28.512)	0.006				
**Regional nodes examined**	No examined nodes	Ref.		0.003	Ref.		0.283
	≤15	2.132 (1.045-4.349)	0.037		1.469 (0.456-4.734)	0.520	

Variable	Category	Univariable analysis			Multivariable analysis		
		HR (95% CI)	*P*	Overall *P*	HR (95% CI)	*P*	Overall *P*
	16-30	2.574 (1.551-4.270)	<0.001		1.805 (0.618-5.272)	0.280	
	>30	1.387 (0.872-2.206)	0.168		1.116 (0.401-3.108)	0.834	
**Diagnosis-to-treatment time**	≤1 month	Ref.		0.408			
	1-3 months	0.790 (0.517-1.207)	0.275				
	3-6 months	0.484 (0.153-1.536)	0.218				
	>6 months	1.095 (0.401-2.994)	0.859				
**Therapy**	ET	Ref.		<0.001	Ref.		0.007
	ET+CRT	10.091 (4.690-21.715)	<0.001		3.788 (1.481-9.685)	0.005	
	G	1.900 (1.173-3.077)	0.009		0.915 (0.314-2.668)	0.871	
	G+CRT	3.274 (1.880-5.702)	<0.001		0.637 (0.192-2.116)	0.461	
**Resection status**	R0 resection	Ref.		0.298			
	Non-R0 resection	1.782 (0.657-4.834)	0.257				

HR, hazard ratio; CI, confidence interval; RNE, regional nodes examined; NE, not estimable because no events occurred in that category; AJCC, American Joint Committee on Cancer; ET, endoscopic therapy; ET+CRT, endoscopic therapy plus chemoradiotherapy; G, gastrectomy; G+CRT, gastrectomy plus chemoradiotherapy. Reference categories are indicated as Ref.

Table S14. Baseline characteristics of patients with lymph node-positive early gastric cancer in the SEER and external validation cohorts

Variable	Category	SEER cohort (n=220)	External cohort (n=144)	P
Age, years	<55	39 (17.7)	46 (31.9)	<0.001
	55-64	49 (22.3)	36 (25.0)	
	65-74	73 (33.2)	50 (34.7)	
	75-84	49 (22.3)	11 (7.6)	
	≥85	10 (4.5)	1 (0.7)	
Sex	Male	126 (57.3)	87 (60.4)	0.552
	Female	94 (42.7)	57 (39.6)	
Race	White	138 (62.7)	1 (0.7)	<0.001
	Black	32 (14.5)	0 (0.0)	
	Other	50 (22.7)	143 (99.3)	
Marital status	Married	131 (59.5)	134 (93.1)	<0.001
	Single	39 (17.7)	7 (4.9)	
	Other	50 (22.7)	3 (2.1)	
Primary site	Cardia	70 (31.8)	18 (12.5)	<0.001
	Pylorus	9 (4.1)	3 (2.1)	
	Other	141 (64.1)	123 (85.4)	
Histology	Adenocarcinoma	186 (84.5)	119 (82.6)	0.338
	Signet ring cell carcinoma	20 (9.1)	19 (13.2)	
	Other	14 (6.4)	6 (4.2)	
Tumor size	≤2 cm	77 (35.0)	55 (38.2)	0.538
	2-3 cm	57 (25.9)	41 (28.5)	
	>3 cm	86 (39.1)	48 (33.3)	
T stage	T1a	36 (16.4)	20 (13.9)	0.522
	T1b	184 (83.6)	124 (86.1)	
N stage	N1	149 (67.7)	84 (58.3)	0.186
	N2	50 (22.7)	43 (29.9)	
	N3	21 (9.5)	17 (11.8)	
AJCC stage	I	110 (50.0)	80 (55.6)	0.579
	II	102 (46.4)	59 (41.0)	
	III	8(3.6)	5 (3.5)	
	IV	0(0.0)	0 (0.0)	
Regional nodes examined	No examined	1 (0.5)	6 (4.2)	<0.001
	Biopsy only	1 (0.5)	0 (0.0)	
	≤15	66 (30.0)	7 (4.9)	
	16-30	116 (52.7)	38 (26.4)	
	>30	36 (16.4)	93 (64.6)	
Therapy	ET	1 (0.5)	0 (0.0)	0.019
	ET+CRT	2 (0.9)	9 (6.2)	
	G	53 (24.1)	27 (18.8)	
	G+CRT	164 (74.5)	108 (75.0)	

Variable	Category	SEER cohort (n=220)	External cohort (n=144)	*P*
Diagnosis-to-treatment time	≤1 month	71 (32.3)	79 (54.9)	<0.001
	1-3 months	132 (60.0)	57 (39.6)	
	3-6 months	14 (6.4)	4 (2.8)	
	>6 months	3 (1.4)	4 (2.8)	

AJCC, American Joint Committee on Cancer; SEER, Surveillance, Epidemiology, and End Results; ET, endoscopic therapy; ET+CRT, endoscopic therapy plus chemoradiotherapy; G, gastrectomy; G+CRT, gastrectomy plus chemoradiotherapy. Values are presented as n (%). Lymph node positivity was defined as N1-N3. P values were calculated using Pearson chi-square tests. Sparse categories should be interpreted cautiously.

Table S15. Baseline characteristics of patients with lymph node-positive early gastric cancer stratified by N stage in the SEER cohort

Variable	Category	N1 (*n*=149)	N2 (*n*=50)	N3 (*n*=21)	*P*
Age, years	<55	24 (16.1)	10 (20.0)	5 (23.8)	0.293
	55-64	32 (21.5)	9 (18.0)	8 (38.1)	
	65-74	48 (32.2)	21 (42.0)	4 (19.0)	
	75-84	38 (25.5)	7 (14.0)	4 (19.0)	
	≥85	7 (4.7)	3 (6.0)	0 (0.0)	
Sex	Male	88 (59.1)	29 (58.0)	9 (42.9)	0.370
	Female	61 (40.9)	21 (42.0)	12 (57.1)	
Race	White	101 (67.8)	24 (48.0)	13 (61.9)	0.103
	Black	21 (14.1)	8 (16.0)	3 (14.3)	
	Other	27 (18.1)	18 (36.0)	5 (23.8)	
Marital status	Married	86 (57.7)	29 (58.0)	16 (76.2)	0.476
	Single	26 (17.4)	11 (22.0)	2 (9.5)	
	Other	37 (24.8)	10 (20.0)	3 (14.3)	
Primary site	Cardia	58 (38.9)	11 (22.0)	1 (4.8)	0.002
	Pylorus	8 (5.4)	1 (2.0)	0 (0.0)	
	Other	83 (55.7)	38 (76.0)	20 (95.2)	
Histology	Adenocarcinoma	131 (87.9)	41 (82.0)	14 (66.7)	0.078
	Signet ring cell carcinoma	9 (6.0)	6 (12.0)	5 (23.8)	
	Other	9 (6.0)	3 (6.0)	2 (9.5)	
Tumor size	≤2 cm	54 (36.2)	20 (40.0)	3 (14.3)	0.064
	2-3 cm	38 (25.5)	15 (30.0)	4 (19.0)	
	>3 cm	57 (38.3)	15 (30.0)	14 (66.7)	
T stage	T1a	22 (14.8)	8 (16.0)	6 (28.6)	0.277
	T1b	127 (85.2)	42 (84.0)	15 (71.4)	
AJCC stage	I	110 (73.8)	0 (0.0)	0 (0.0)	<0.001
	II	39 (26.2)	44 (88.0)	19 (90.5)	
	III	0 (0.0)	6 (12.0)	2(9.5)	
	IV	0 (0.0)	0 (0.0)	0(0.0)	
Regional nodes examined	No examined	1 (0.7)	0 (0.0)	0 (0.0)	0.352
	Biopsy only	1 (0.7)	0 (0.0)	0 (0.0)	
	≤15	50 (33.6)	13 (26.0)	3 (14.3)	
	16-30	78 (52.3)	27 (54.0)	11 (52.4)	
	>30	19 (12.8)	10 (20.0)	7 (33.3)	
Therapy	ET	1 (0.7)	0 (0.0)	0 (0.0)	0.469
	ET+CRT	2 (1.3)	0 (0.0)	0 (0.0)	
	G	35 (23.5)	16 (32.0)	2 (9.5)	
	G+CRT	111 (74.5)	34 (68.0)	19 (90.5)	
Diagnosis-to-treatment time	≤1 month	44 (29.5)	15 (30.0)	12 (57.1)	0.166
	1-3 months	91 (61.1)	33 (66.0)	8 (38.1)	

Variable	Category	N1 (*n*=149)	N2 (*n*=50)	N3 (*n*=21)	*P*
	3-6 months	12 (8.1)	1 (2.0)	1 (4.8)	
	>6 months	2 (1.3)	1 (2.0)	0 (0.0)	

AJCC, American Joint Committee on Cancer; SEER, Surveillance, Epidemiology, and End Results; ET, endoscopic therapy; ET+CRT, endoscopic therapy plus chemoradiotherapy; G, gastrectomy; G+CRT, gastrectomy plus chemoradiotherapy. N stage was the stratification variable and was therefore not repeated as a table row. Values are presented as n (%). Lymph node positivity was defined as N1-N3. P values were calculated using Pearson chi-square tests.

Table S16. Baseline characteristics of patients with lymph node-positive early gastric cancer stratified by N stage in the external validation cohort

Variable	Category	N1 (*n*=84)	N2 (*n*=43)	N3 (*n*=17)	*P*
Age, years	<55	27 (32.1)	13 (30.2)	6 (35.3)	0.653
	55-64	16 (19.0)	14 (32.6)	6 (35.3)	
	65-74	34 (40.5)	12 (27.9)	4 (23.5)	
	75-84	6 (7.1)	4 (9.3)	1 (5.9)	
	≥85	1 (1.2)	0 (0.0)	0 (0.0)	
Sex	Male	51 (60.7)	27 (62.8)	9 (52.9)	0.778
	Female	33 (39.3)	16 (37.2)	8 (47.1)	
Race	White	1 (1.2)	0 (0.0)	0 (0.0)	0.698
	Black	0 (0.0)	0 (0.0)	0 (0.0)	
	Other	83 (98.8)	43 (100.0)	17 (100.0)	
Marital status	Married	78 (92.9)	39 (90.7)	17 (100.0)	0.783
	Single	4 (4.8)	3 (7.0)	0 (0.0)	
	Other	2 (2.4)	1 (2.3)	0 (0.0)	
Primary site	Cardia	13 (15.5)	4 (9.3)	1 (5.9)	0.418
	Pylorus	2 (2.4)	0 (0.0)	1 (5.9)	
	Other	69 (82.1)	39 (90.7)	15 (88.2)	
Histology	Adenocarcinoma	70 (83.3)	37 (86.0)	12 (70.6)	0.158
	Signet ring cell carcinoma	11 (13.1)	3 (7.0)	5 (29.4)	
	Other	3 (3.6)	3 (7.0)	0 (0.0)	
Tumor size	≤2 cm	29 (34.5)	22 (51.2)	4 (23.5)	0.228
	2-3 cm	24 (28.6)	10 (23.3)	7 (41.2)	
	>3 cm	31 (36.9)	11 (25.6)	6 (35.3)	
T stage	T1a	11 (13.1)	6 (14.0)	3 (17.6)	0.885
	T1b	73 (86.9)	37 (86.0)	14 (82.4)	
AJCC stage	I	80 (95.2)	0 (0.0)	0 (0.0)	<0.001
	II	4 (4.8)	43 (100.0)	12 (70.6)	
	III	0 (0.0)	0 (0.0)	5 (29.4)	
	IV	0 (0.0)	0 (0.0)	0 (0.0)	
Regional nodes examined	No examined	6 (7.1)	0 (0.0)	0 (0.0)	0.406
	Biopsy only	0 (0.0)	0 (0.0)	0 (0.0)	
	≤15	5 (6.0)	1 (2.3)	1 (5.9)	
	16-30	20 (23.8)	12 (27.9)	6 (35.3)	
	>30	53 (63.1)	30 (69.8)	10 (58.8)	
Therapy	ET	0 (0.0)	0 (0.0)	0 (0.0)	0.939
	ET+CRT	6 (7.1)	2 (4.7)	1 (5.9)	
	G	16 (19.0)	7 (16.3)	4 (23.5)	
	G+CRT	62 (73.8)	34 (79.1)	12 (70.6)	
Diagnosis-to-treatment time	≤1 month	46 (54.8)	21 (48.8)	12 (70.6)	0.168
	1-3 months	36 (42.9)	17 (39.5)	4 (23.5)	

Variable	Category	N1 (*n*=84)	N2 (*n*=43)	N3 (*n*=17)	*P*
	3-6 months	2 (2.4)	2 (4.7)	0 (0.0)	
	>6 months	0 (0.0)	3 (7.0)	1 (5.9)	

AJCC, American Joint Committee on Cancer; CRT, chemoradiotherapy. N stage was the stratification variable and was therefore not repeated as a table row; ET, endoscopic therapy; ET+CRT, endoscopic therapy plus chemoradiotherapy; G, gastrectomy; G+CRT, gastrectomy plus chemoradiotherapy. Values are presented as n (%). Lymph node positivity was defined as N1-N3. P values were calculated using Pearson chi-square tests. Sparse categories should be interpreted cautiously.

Table S17. Five-year OS and CSS according to N stage

Cohort	Endpoint	N stage	*n*	Events	5-year survival, % (95% CI)
SEER development cohort	CSS	N1	149	17	87.2 (80.1-91.9)
		N2	50	12	71.9 (54.2-83.7)
		N3	21	4	66.0 (25.8-88.1)
	OS	N1	149	31	73.6 (60.8-82.8)
		N2	50	15	66.7 (49.6-79.1)
		N3	21	5	60.5 (24.3-83.7)
External validation cohort	CSS	N1	84	11	86.4 (76.7-92.2)
		N2	43	8	80.6 (64.9-89.8)
		N3	17	6	63.3 (35.8-81.6)
	OS	N1	84	19	77.4 (66.9-84.9)
		N2	43	11	74.4 (58.6-84.9)
		N3	17	7	58.8 (32.5-77.8)

CSS, cancer-specific survival; OS, overall survival; CI, confidence interval; SEER, Surveillance, Epidemiology, and End Results. Survival estimates were obtained using the Kaplan-Meier method at 60 months.

Table S18. Cox and log-rank analyses according to N stage

Cohort	Endpoint	Comparison	HR (95% CI)	Cox P	Log-rank *P*
SEER development cohort	CSS	N2 vs N1	2.25 (1.07-4.72)	0.032	0.027
		N3 vs N1	1.71 (0.58-5.09)	0.334	0.326
		N3 vs N2	0.80 (0.26-2.48)	0.693	0.692
	OS	N2 vs N1	1.51 (0.82-2.81)	0.188	0.183
		N3 vs N1	1.20 (0.47-3.08)	0.709	0.708
		N3 vs N2	0.80 (0.29-2.19)	0.658	0.657
External validation cohort	CSS	N2 vs N1	1.51 (0.61-3.75)	0.376	0.372
		N3 vs N1	3.39 (1.25-9.18)	0.016	0.010
		N3 vs N2	2.22 (0.77-6.40)	0.141	0.131
	OS	N2 vs N1	1.20 (0.57-2.52)	0.631	0.629
		N3 vs N1	2.33 (0.98-5.56)	0.056	0.048
		N3 vs N2	1.89 (0.73-4.88)	0.189	0.180

CSS, cancer-specific survival; OS, overall survival; HR, hazard ratio; CI, confidence interval; SEER, Surveillance, Epidemiology, and End Results.

Table S19. Chemotherapy regimen distribution according to nodal stage among node-positive patients receiving gastrectomy plus chemoradiotherapy in the external validation cohort

N stage	*n*	S-1	SOX	XELOX	Other	Overall *P*
N1	62	21 (33.9%)	16 (25.8%)	8 (12.9%)	17 (27.4%)	0.075
N2	34	6 (17.6%)	15 (44.1%)	8 (23.5%)	5 (14.7%)	
N3	12	6 (50.0%)	2 (16.7%)	3 (25.0%)	1 (8.3%)	

Values are *n* (% within each N stage). S-1, an oral fluoropyrimidine derivative (tegafur/gimeracil/oteracil); SOX, S-1 plus oxaliplatin; XELOX, capecitabine plus oxaliplatin. Other includes TS, DOS, FOLFOX, DS, and CAP.

Table S20. Survival outcomes according to chemotherapy regimen among node-positive patients receiving gastrectomy plus chemoradiotherapy in the external validation cohort

Regimen	*n*	OS deaths	1-year OS (95% CI)	3-year OS (95% CI)	5-year OS (95% CI)	CSS deaths	1-year CSS (95% CI)	3-year CSS (95% CI)	5-year CSS (95% CI)
S-1	33	7	87.9% (70.9-95.3)	84.8% (67.4-93.4)	78.8% (60.6-89.3)	4	90.9% (74.4-97.0)	90.9% (74.4-97.0)	87.5% (70.1-95.1)
SOX	33	5	97.0% (80.4-99.6)	90.9% (74.4-97.0)	84.8% (67.4-93.4)	3	100.0% (100.0-100.0)	93.8% (77.3-98.4)	90.6% (73.7-96.9)
XELOX	19	7	89.5% (64.1-97.3)	68.4% (42.8-84.4)	63.2% (37.9-80.4)	3	94.7% (68.1-99.2)	83.2% (56.4-94.3)	83.2% (56.4-94.3)
Other	23	5	100.0% (100.0-100.0)	78.3% (55.4-90.3)	78.3% (55.4-90.3)	4	100.0% (100.0-100.0)	82.6% (60.1-93.1)	82.6% (60.1-93.1)

CSS, cancer-specific survival; OS, overall survival; CI, confidence interval; SOX, S-1 plus oxaliplatin; XELOX, capecitabine plus oxaliplatin; S-1, an oral fluoropyrimidine derivative (tegafur/gimeracil/oteracil).

Table S21. Univariable Cox Regression Analysis for Cancer-Specific Survival

Variable	Category	HR (95% CI)	*P*	Overall *P*
Age, years	<55	Ref.		<0.001
	55-64	1.473 (0.645-3.364)	0.358	
	65-74	1.843 (0.856-3.964)	0.118	
	75-84	4.163 (1.990-8.706)	<0.001	
	≥85	3.264 (1.288-8.275)	0.013	
Sex	Female	Ref.		0.043
	Male	1.453 (1.003-2.105)	0.048	
Race	White	Ref.		0.024
	Black	1.080 (0.637-1.830)	0.776	
	Other	0.527 (0.315-0.881)	0.015	
Marital status	Married	Ref.		0.084
	Single	0.885 (0.541-1.447)	0.626	
	Other	1.523 (1.020-2.273)	0.040	
Primary site	Cardia	Ref.		0.169
	Pylorus	0.557 (0.174-1.779)	0.323	
	Other	0.732 (0.517-1.036)	0.079	
Histology	Adenocarcinoma	Ref.		0.328
	Signet ring cell carcinoma	0.705 (0.404-1.227)	0.216	
	Other	0.670 (0.247-1.816)	0.431	
Tumor size	≤2 cm	Ref.		<0.001
	2-3 cm	1.379 (0.861-2.209)	0.181	
	>3 cm	2.191 (1.485-3.231)	<0.001	
T stage	T1a	Ref.		<0.001
	T1b	2.725 (1.845-4.023)	<0.001	
N stage	N0	Ref.		<0.001
	N1	1.816 (1.086-3.037)	0.023	
	N2	4.174 (2.293-7.598)	<0.001	
	N3	3.173 (1.168-8.622)	0.024	
AJCC stage	I	Ref.		<0.001
	II	2.666 (1.601-4.440)	<0.001	
	III	6.209 (1.972-19.546)	0.002	
Regional nodes examined	No examined	Ref.		<0.001

Variable	Category	HR (95% CI)	*P*	Overall *P*
	Biopsy only	1.761 (0.241-12.859)	0.577	
	≤15	1.728 (1.127-2.649)	0.012	
	16-30	1.129 (0.717-1.777)	0.601	
	>30	0.285 (0.101-0.803)	0.018	
Therapy	ET	Ref.		<0.001
	ET+CRT	6.314 (2.981-13.370)	<0.001	
	G	1.691 (1.009-2.835)	0.046	
	G+CRT	2.710 (1.521-4.829)	<0.001	
Diagnosis-to-treatment time	≤1 month	Ref.		0.823
	1-3 months	0.899 (0.625-1.292)	0.564	
	3-6 months	0.910 (0.488-1.698)	0.767	
	>6 months	1.411 (0.512-3.892)	0.506	

HR, hazard ratio; CI, confidence interval; CSS, cancer-specific survival; CRT, chemoradiotherapy; AJCC, American Joint Committee on Cancer; ET, endoscopic therapy; ET+CRT, endoscopic therapy plus chemoradiotherapy; G, gastrectomy; G+CRT, gastrectomy plus chemoradiotherapy. Reference-category HRs were set to 1.000. Overall P values were obtained from likelihood-ratio tests.

Table S22. Comparison of Variable Selection Methods and Final Model

Variable	Univariate P	Univariate P<0.10	LASSO alpha_min	LASSO alpha_1se	Boruta status	BSR final model
Age	<0.001	TRUE	TRUE	TRUE	Confirmed	TRUE
Sex	0.043	TRUE	TRUE	TRUE	Rejected	TRUE
Race	0.024	TRUE	TRUE	TRUE	Rejected	TRUE
Marital Status	0.083	TRUE	TRUE	FALSE	Rejected	FALSE
Primary Site	0.169	FALSE	TRUE	FALSE	Rejected	FALSE
Histology	0.328	FALSE	FALSE	FALSE	Rejected	FALSE
Size	<0.001	TRUE	TRUE	TRUE	Rejected	FALSE
T	<0.001	TRUE	TRUE	TRUE	Confirmed	TRUE
N	<0.001	TRUE	TRUE	TRUE	Rejected	TRUE
AJCC	<0.001	TRUE	TRUE	TRUE	Rejected	FALSE
RNE	<0.001	TRUE	TRUE	TRUE	Confirmed	TRUE
Therapy	<0.001	TRUE	TRUE	TRUE	Rejected	TRUE
DTTT	0.823	FALSE	TRUE	FALSE	Rejected	FALSE

RNE, regional lymph nodes examined (RNE); DTTT, diagnosis-to-treatment time; BSR, best-subset regression; LASSO, least absolute shrinkage and selection operator; AJCC, American Joint Committee on Cancer

Table S23. Multivariable Cox Regression Analysis for Cancer-Specific Survival

**Variable**	**Category**	**Adjusted HR (95% CI)**	***P***
Age, years	<55	Ref.	
	55-64	1.390 (0.605-3.196)	0.438
	65-74	1.589 (0.728-3.468)	0.244
	75-84	3.701 (1.740-7.874)	<0.001
	≥85	2.773 (1.048-7.337)	0.040
Sex	Female	Ref.	
	Male	1.409 (0.966-2.054)	0.075
Race	White	Ref.	
	Black	1.060 (0.620-1.811)	0.831
	Other	0.533 (0.317-0.896)	0.018
T stage	T1a	Ref.	
	T1b	1.963 (1.294-2.977)	0.002
N stage	N0	Ref.	
	N1	1.417 (0.743-2.701)	0.290
	N2	4.324 (2.096-8.920)	<0.001
	N3	3.642 (1.176-11.282)	0.025
Regional nodes examined	No examined	Ref.	
	Biopsy only	1.236 (0.164-9.313)	0.837
	≤15	1.087 (0.500-2.362)	0.834
	16-30	0.682 (0.304-1.530)	0.353
	>30	0.225 (0.066-0.767)	0.017
Therapy	ET	Ref.	
	ET+CRT	4.157 (1.918-9.009)	<0.001
	G	1.968 (0.837-4.632)	0.121
	G+CRT	2.078 (0.787-5.490)	0.140

HR, hazard ratio; CI, confidence interval; CSS, cancer-specific survival; ET, endoscopic therapy; ET+CRT, endoscopic therapy plus chemoradiotherapy; G, gastrectomy; G+CRT, gastrectomy plus chemoradiotherapy; CRT, chemoradiotherapy.

Figure S1

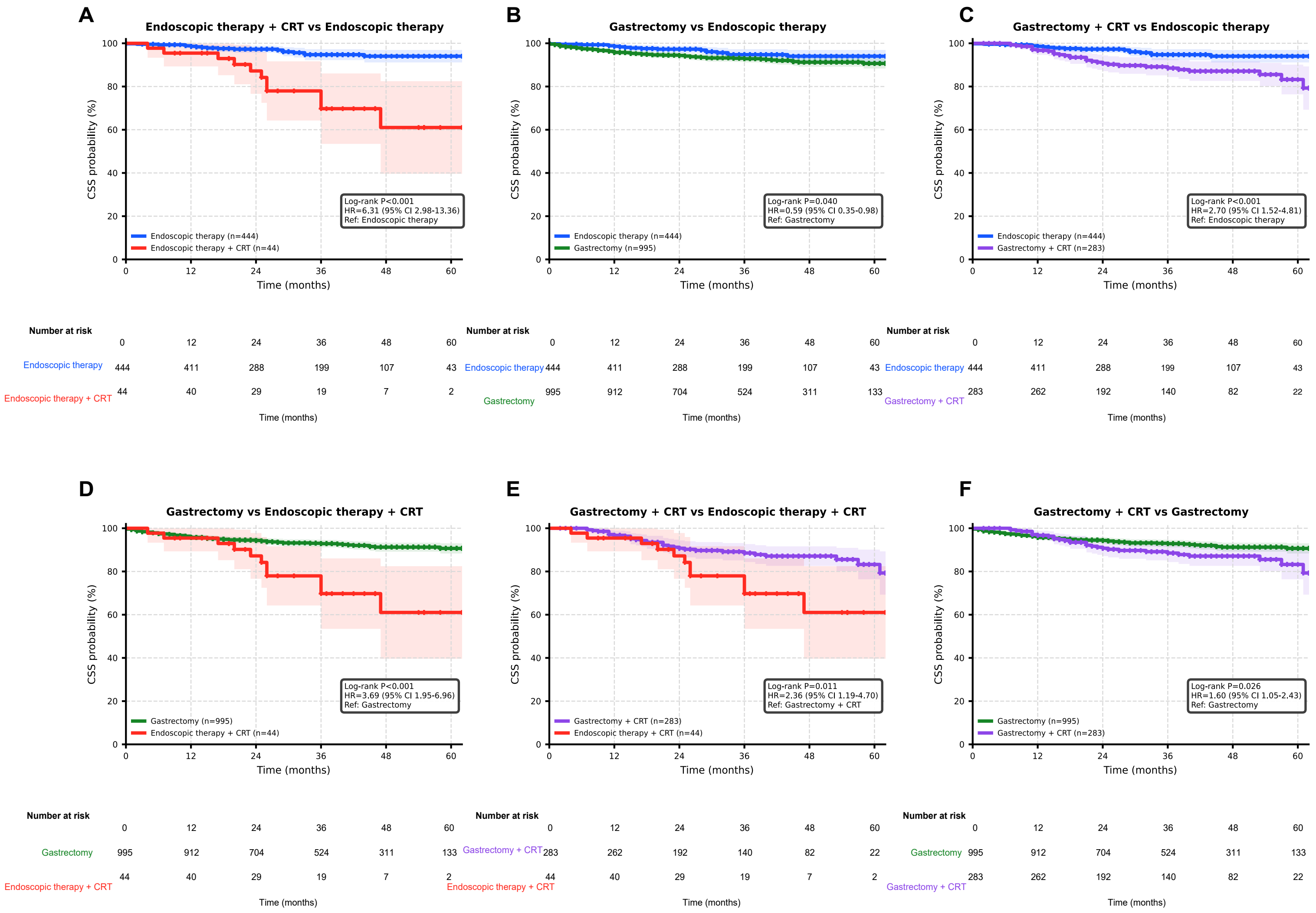

Figure S1. Unadjusted Kaplan-Meier curves comparing different treatments for cancer-specific survival (CSS) in early gastric cancer. Survival outcomes of different treatments: endoscopic therapy + CRT and endoscopic therapy (A), endoscopic therapy and gastrectomy (B), endoscopic therapy and gastrectomy + CRT (C), endoscopic therapy + CRT and gastrectomy (D), endoscopic therapy + CRT and gastrectomy + CRT (E), and gastrectomy + CRT and gastrectomy (F). Hazard ratios (HR) with 95% confidence intervals and log-rank P values are shown in each panel. CSS, cancer-specific survival. CRT, chemoradiotherapy.

Figure S2

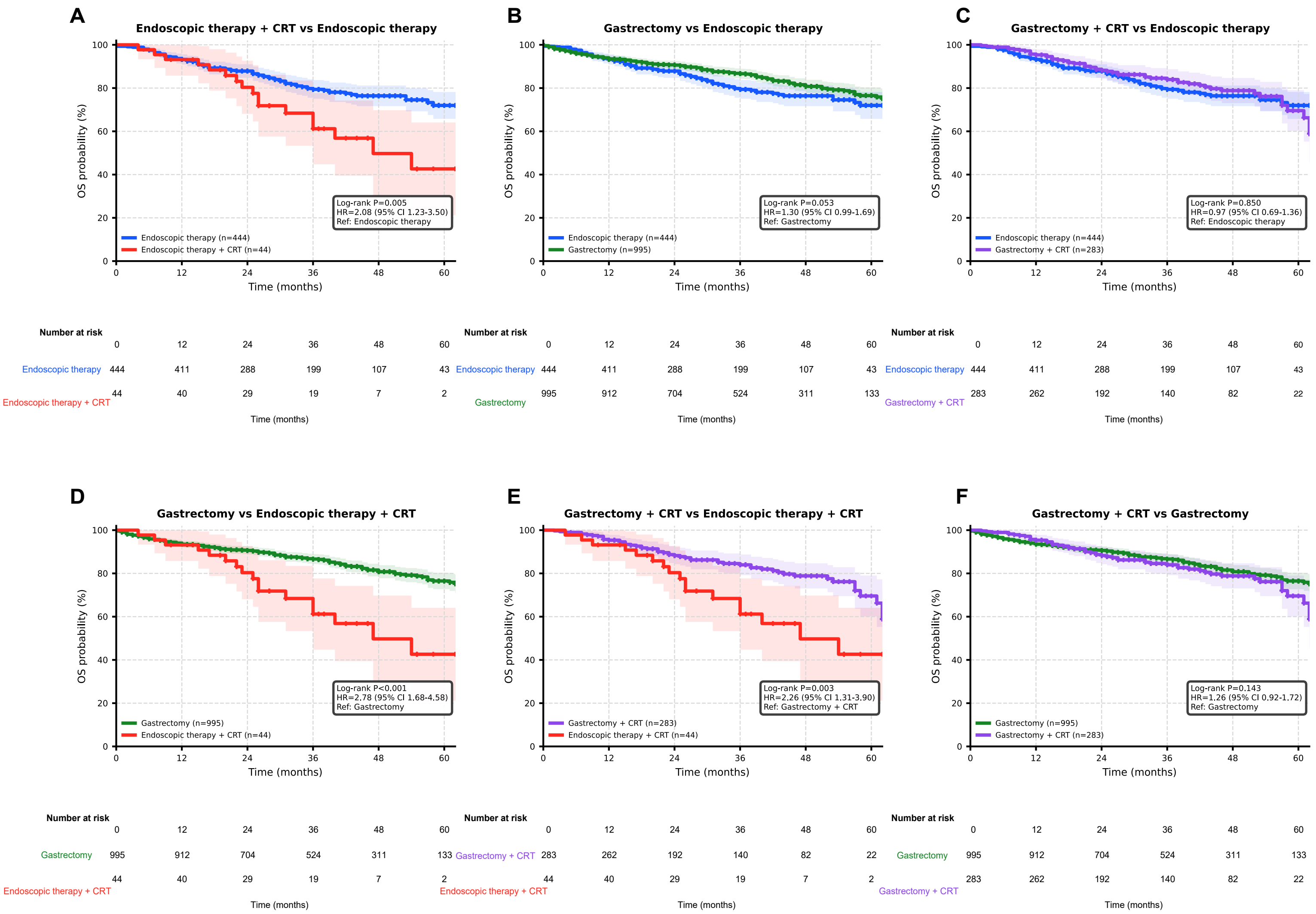

Figure S2. Unadjusted Kaplan-Meier curves comparing different treatments for overall survival (OS) in early gastric cancer. Survival outcomes of different treatments: endoscopic therapy + CRT and endoscopic therapy (A), endoscopic therapy and gastrectomy (B), endoscopic therapy and gastrectomy + CRT (C), endoscopic therapy + CRT and gastrectomy (D), endoscopic therapy + CRT and gastrectomy + CRT (E), and gastrectomy + CRT and gastrectomy (F). Hazard ratios (HR) with 95% confidence intervals and log-rank P values are shown in each panel. OS, overall survival. CRT, chemoradiotherapy.

Figure S3

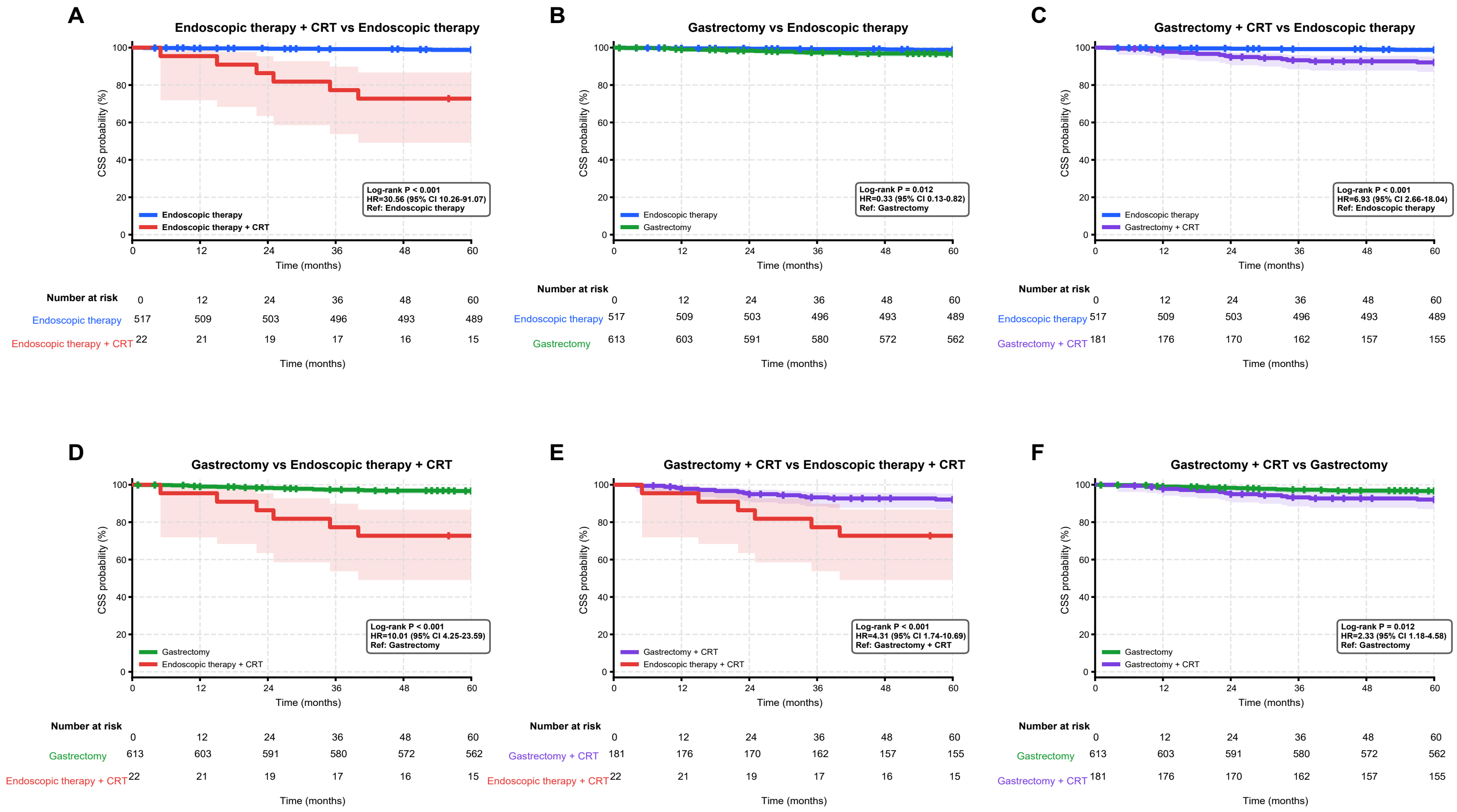

Figure S3. Unadjusted Kaplan-Meier curves comparing different treatments for cancer-specific survival (CSS) in the external validation cohort. Survival outcomes of different treatments: endoscopic therapy + CRT and endoscopic therapy (A), endoscopic therapy and gastrectomy (B), endoscopic therapy and gastrectomy + CRT (C), endoscopic therapy + CRT and gastrectomy (D), endoscopic therapy + CRT and gastrectomy + CRT (E), and gastrectomy + CRT and gastrectomy (F). CSS, cancer-specific survival. CRT, chemoradiotherapy.

Figure S4

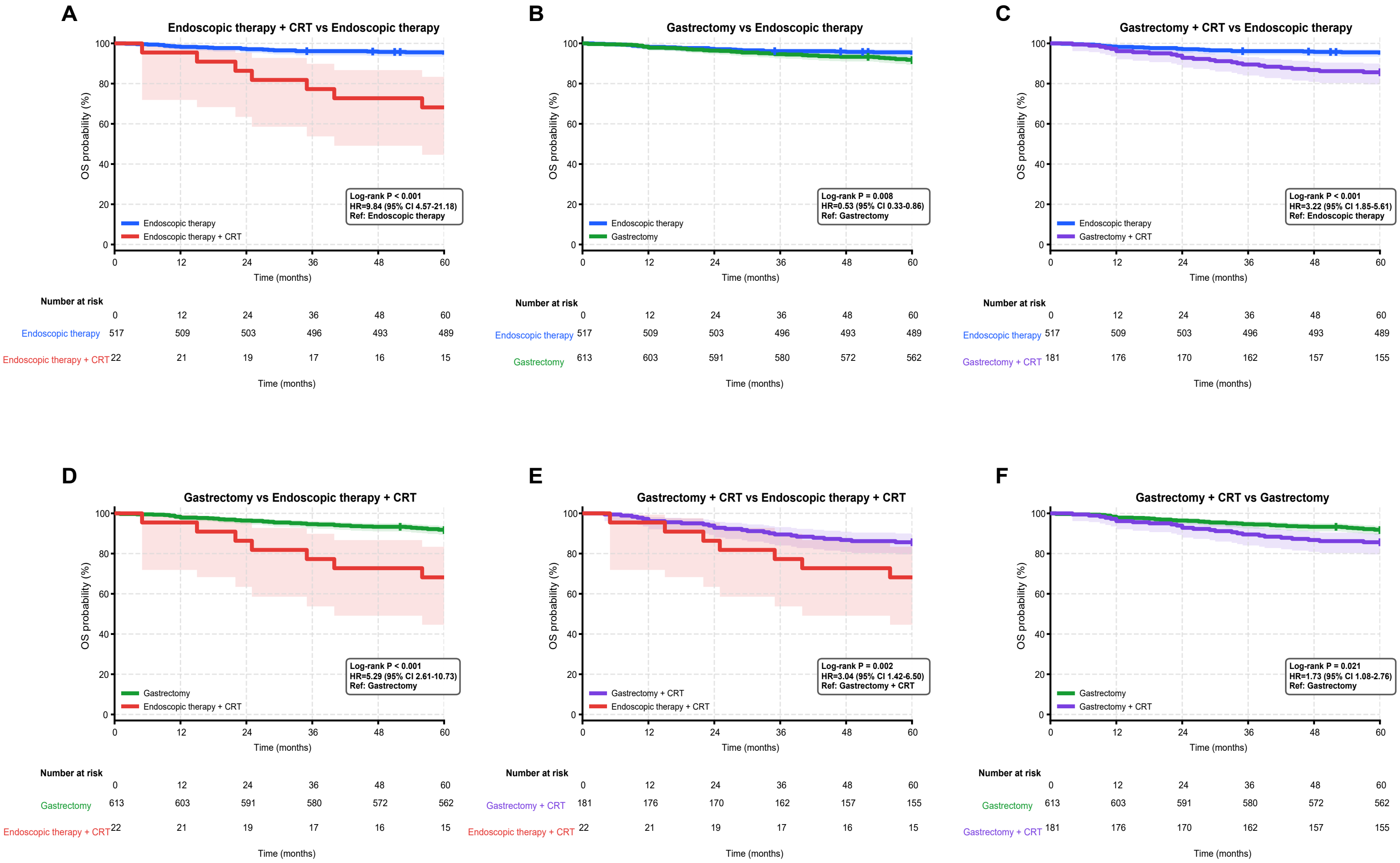

Figure S4. Unadjusted Kaplan-Meier curves comparing different treatments for overall survival (OS) in the external validation cohort. Survival outcomes of different treatments: endoscopic therapy + CRT and endoscopic therapy (A), endoscopic therapy and gastrectomy (B), endoscopic therapy and gastrectomy + CRT (C), endoscopic therapy + CRT and gastrectomy (D), endoscopic therapy + CRT and gastrectomy + CRT (E), and gastrectomy + CRT and gastrectomy (F). OS, overall survival. CRT, chemoradiotherapy.

Figure S5

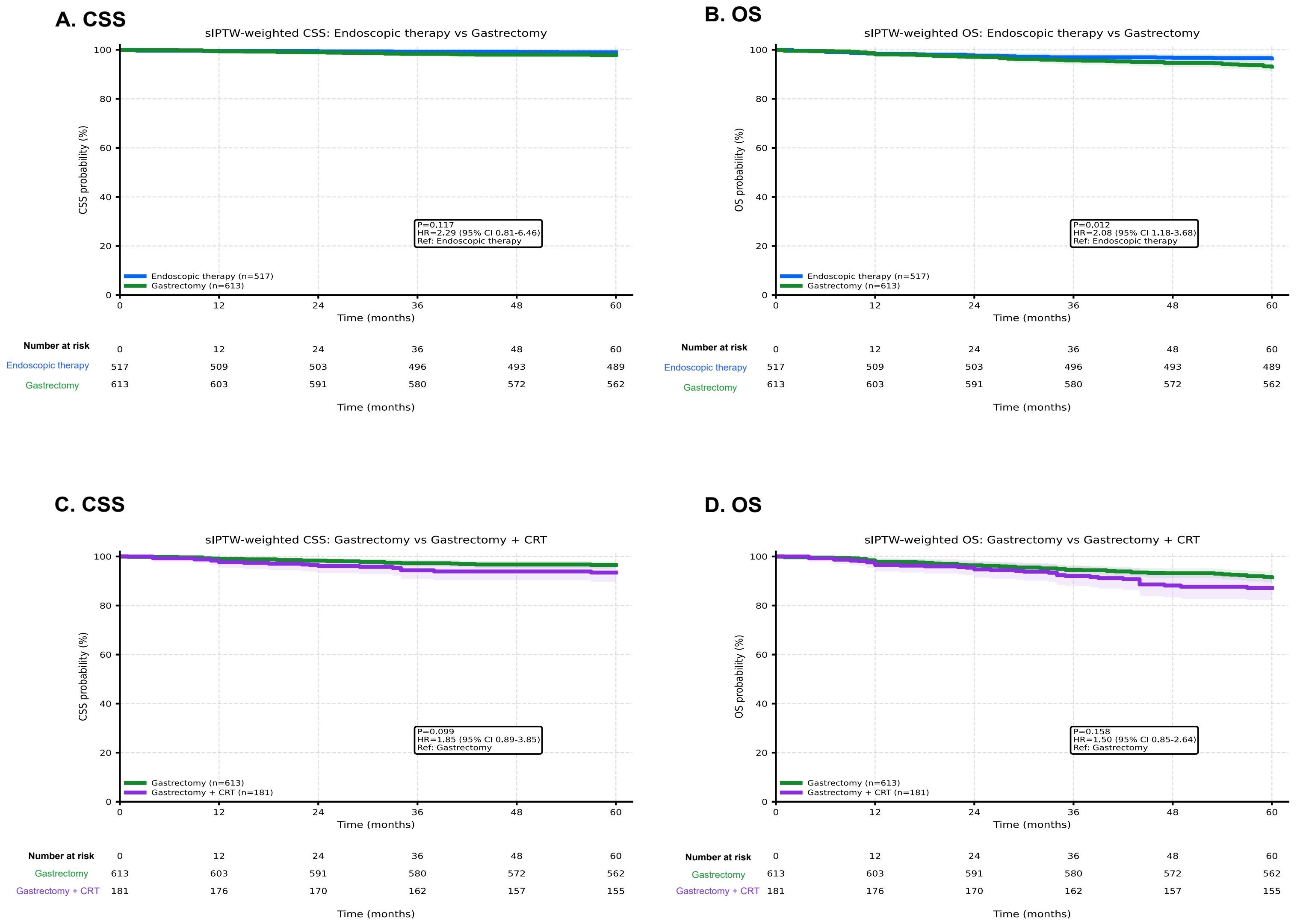

Figure S5. After adjustment using sIPTW, Kaplan-Meier curves were used to compare cancer-specific survival (CSS) and overall survival (OS) outcomes among different treatments for early gastric cancer in the external validation cohort. Survival outcomes of different treatments: endoscopic therapy and gastrectomy for CSS (A) and OS (B), and gastrectomy + CRT and gastrectomy for CSS (C) and OS (D). sIPTW, stabilized inverse probability of treatment weighting; CSS, cancer-specific survival; OS, overall survival; CRT, chemoradiotherapy.

Figure S6

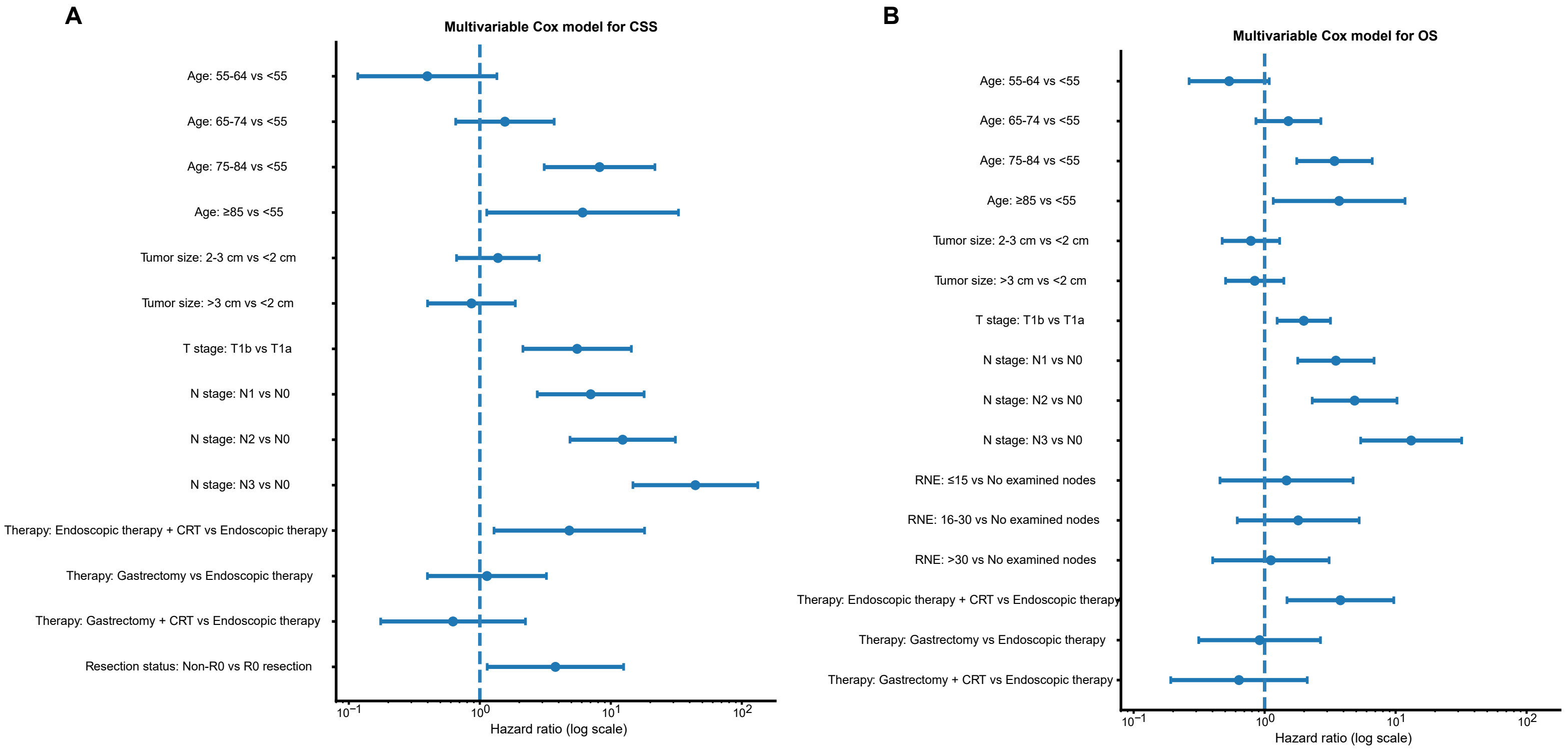

Figure S6. Multivariable Cox proportional hazards analyses in the external validation cohort. (A) Forest plot for cancer-specific survival (CSS). The multivariable model included age, tumor size, T stage, N stage, therapy, and resection status. (B) Forest plot for overall survival (OS). The multivariable model included age, tumor size, T stage, N stage, regional lymph nodes examined (RNE), and therapy.

Figure S7

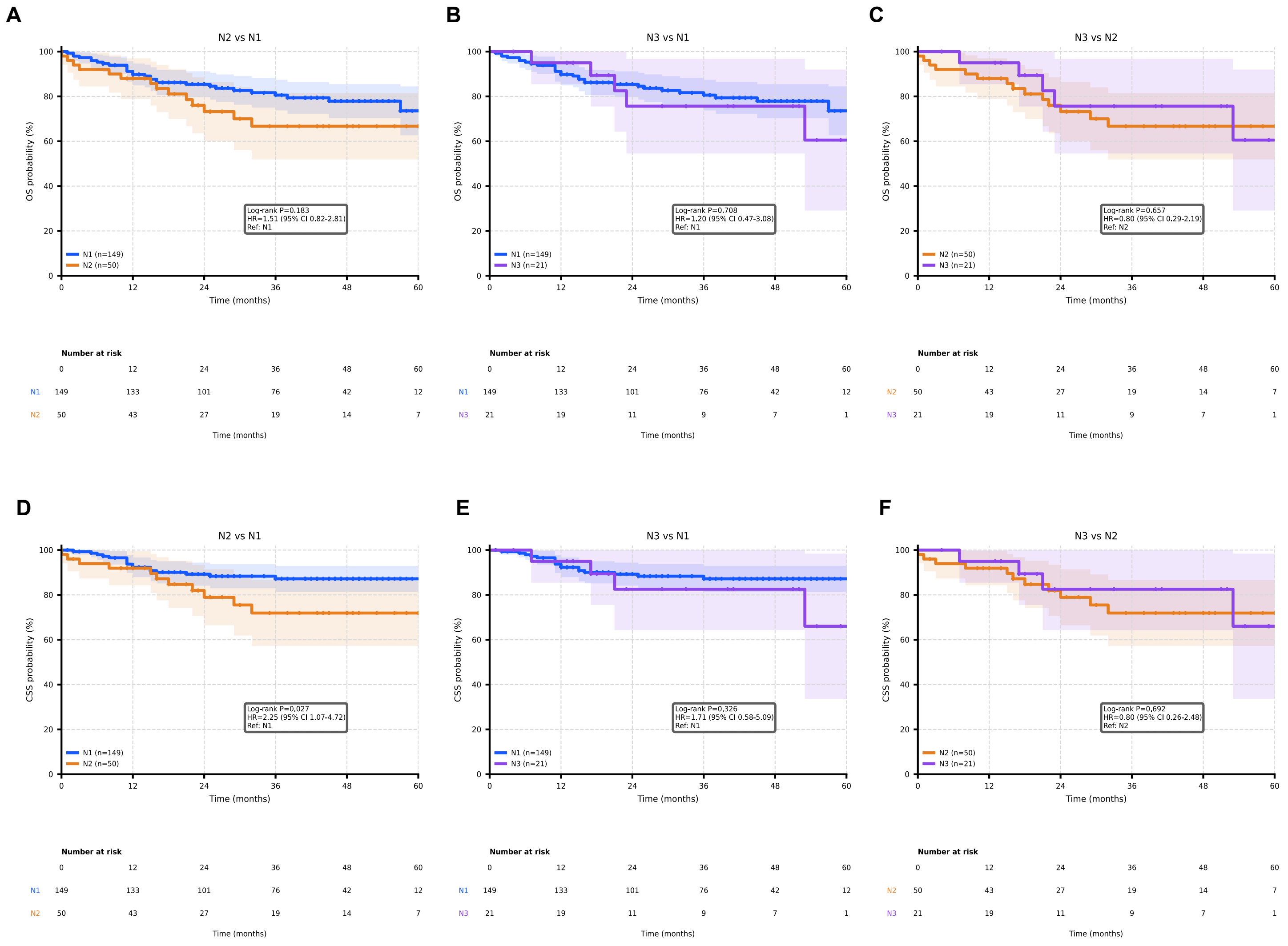

Figure S7. Kaplan-Meier curves by N stage for overall survival (OS) and cancer-specific survival (CSS) in the development cohort (SEER). Panels A–C show OS and panels D–F show CSS, presenting pairwise comparisons among nodal categories N1, N2, and N3. OS, overall survival; CSS, cancer-specific survival.

Figure S8

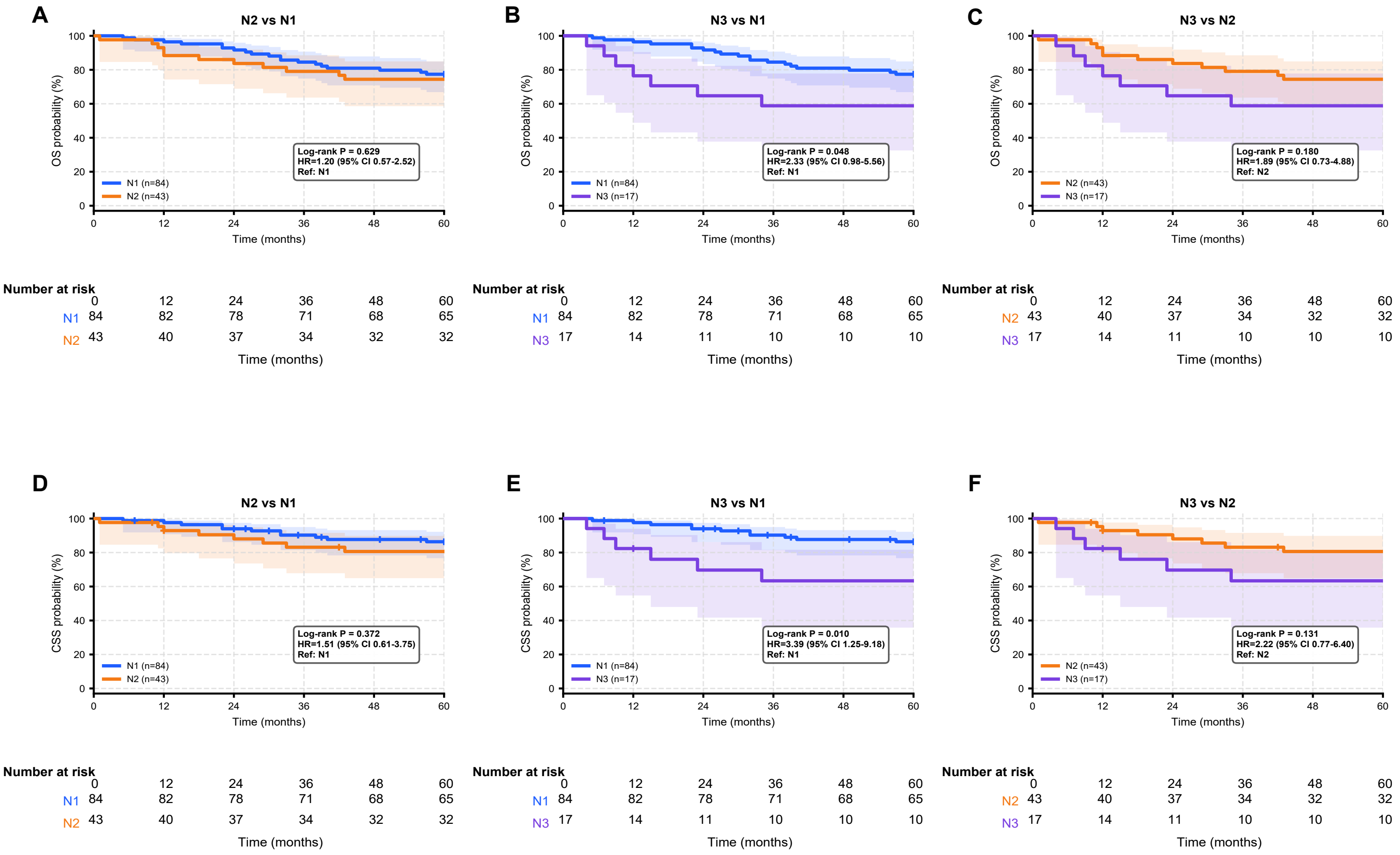

Figure S8. Kaplan-Meier curves by N stage for overall survival (OS) and cancer-specific survival (CSS) in the external validation cohort. Panels A–C show OS and panels D–F show CSS, presenting pairwise comparisons among nodal categories N0, N1, N2, and N3. OS, overall survival; CSS, cancer-specific survival.

Figure S9

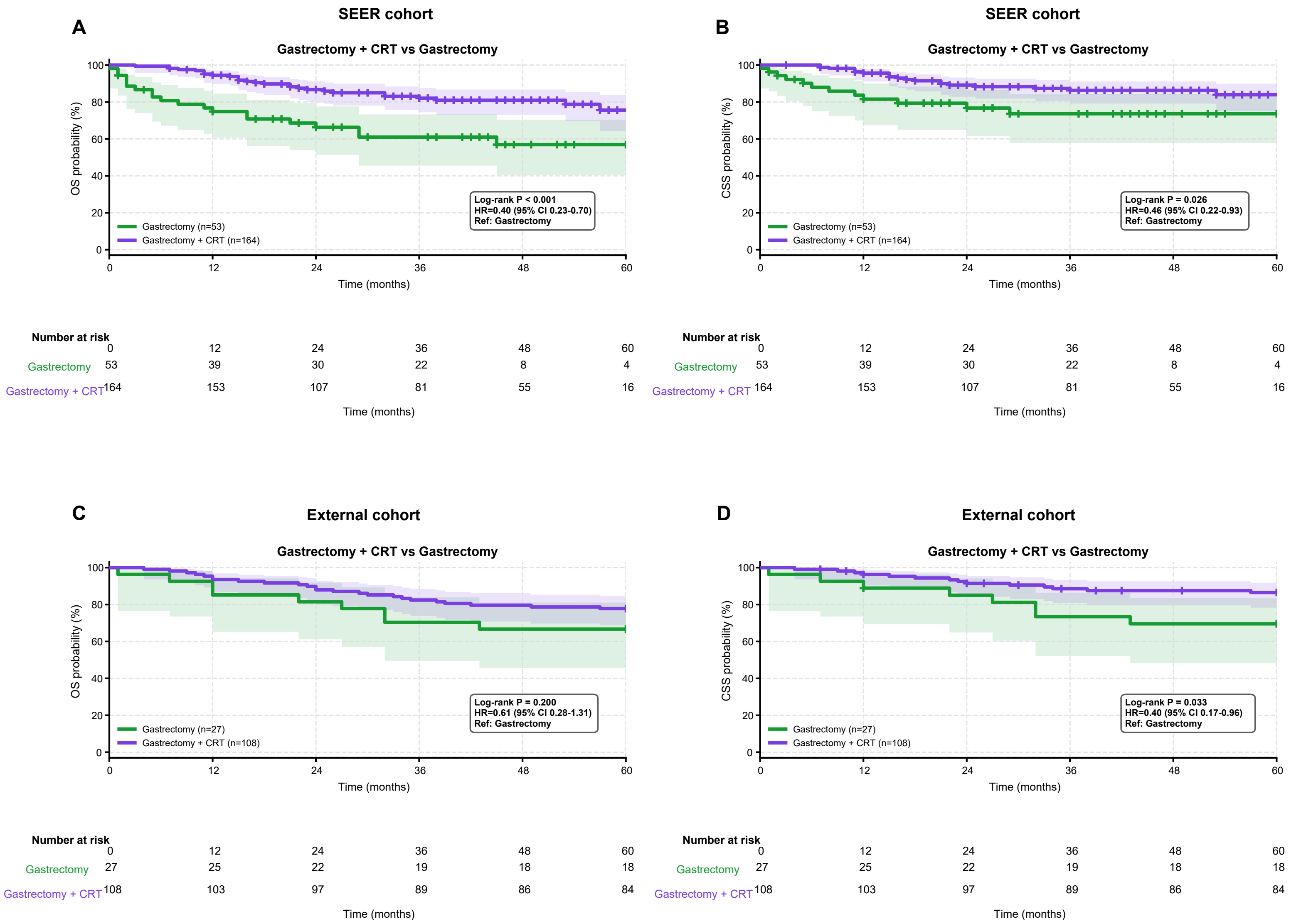

Figure S9. Kaplan-Meier curves comparing gastrectomy combined with chemoradiotherapy (G + CRT) versus gastrectomy alone for cancer-specific survival (CSS) and overall survival (OS) in the node-positive subgroup. The development cohort (SEER) is shown in panels A (CSS) and B (OS), and the external validation cohort in panels C (CSS) and D (OS). CSS, cancer-specific survival; OS, overall survival; CRT, chemoradiotherapy.

Figure S10

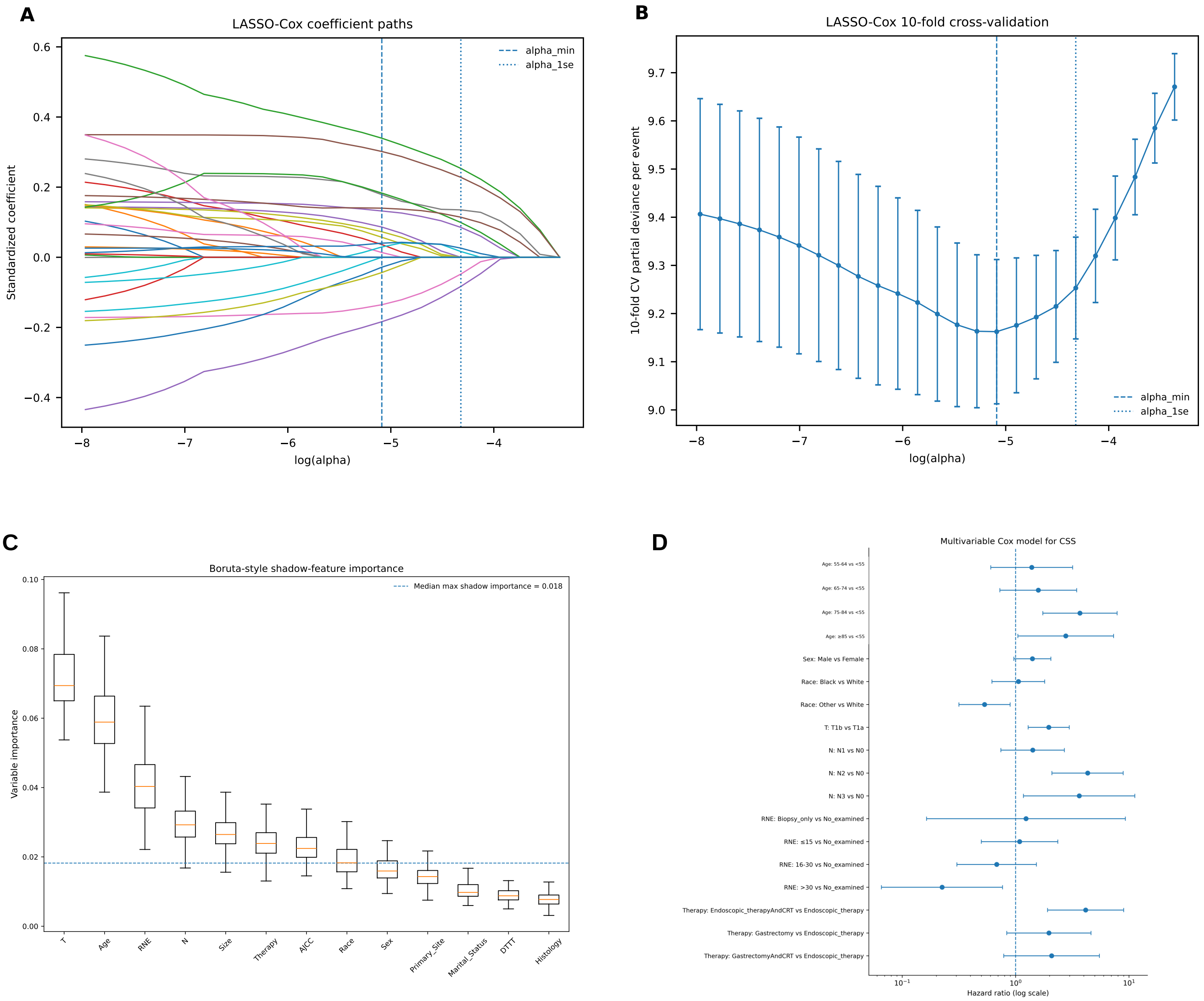

Figure S10. Variable selection using LASSO regression and Boruta analysis, and the final multivariable Cox regression model for predicting cancer-specific survival (CSS) in early gastric cancer. (A) Tenfold cross-validation for tuning parameter (α) selection in the LASSO model. The optimal α under the minimum-error criterion (α_min = 0.006174) and the 1-SE criterion (α_1se = 0.013301) are indicated by the dashed vertical lines. (B) LASSO coefficient curves. Under the more conservative α_1se, 9 predictors with non-zero coefficients are retained—age, sex, race, tumor size, T stage, N stage, AJCC stage, regional lymph nodes examined (RNE) and therapy; under α_min, 12 candidate variables are retained except histology. (C) Boruta algorithm for variable importance. Age, T stage, and the number of regional lymph nodes examined (RNE) were confirmed as important variables, whereas the remaining candidate variables were rejected.(D) Forest plot of the final multivariable Cox regression model. Seven variables (age, sex, race, T stage, N stage, RNE, and therapy) are included; older age, advanced T stage, advanced N stage, insufficient RNE, and endoscopic therapy combined with CRT are independent risk factors for CSS. CSS, cancer-specific survival; RNE, regional lymph nodes examined; DTTT, diagnosis-to-treatment time; LASSO, least absolute shrinkage and selection operator; AJCC, American Joint Committee on Cancer; CRT, chemoradiotherapy

Figure S11

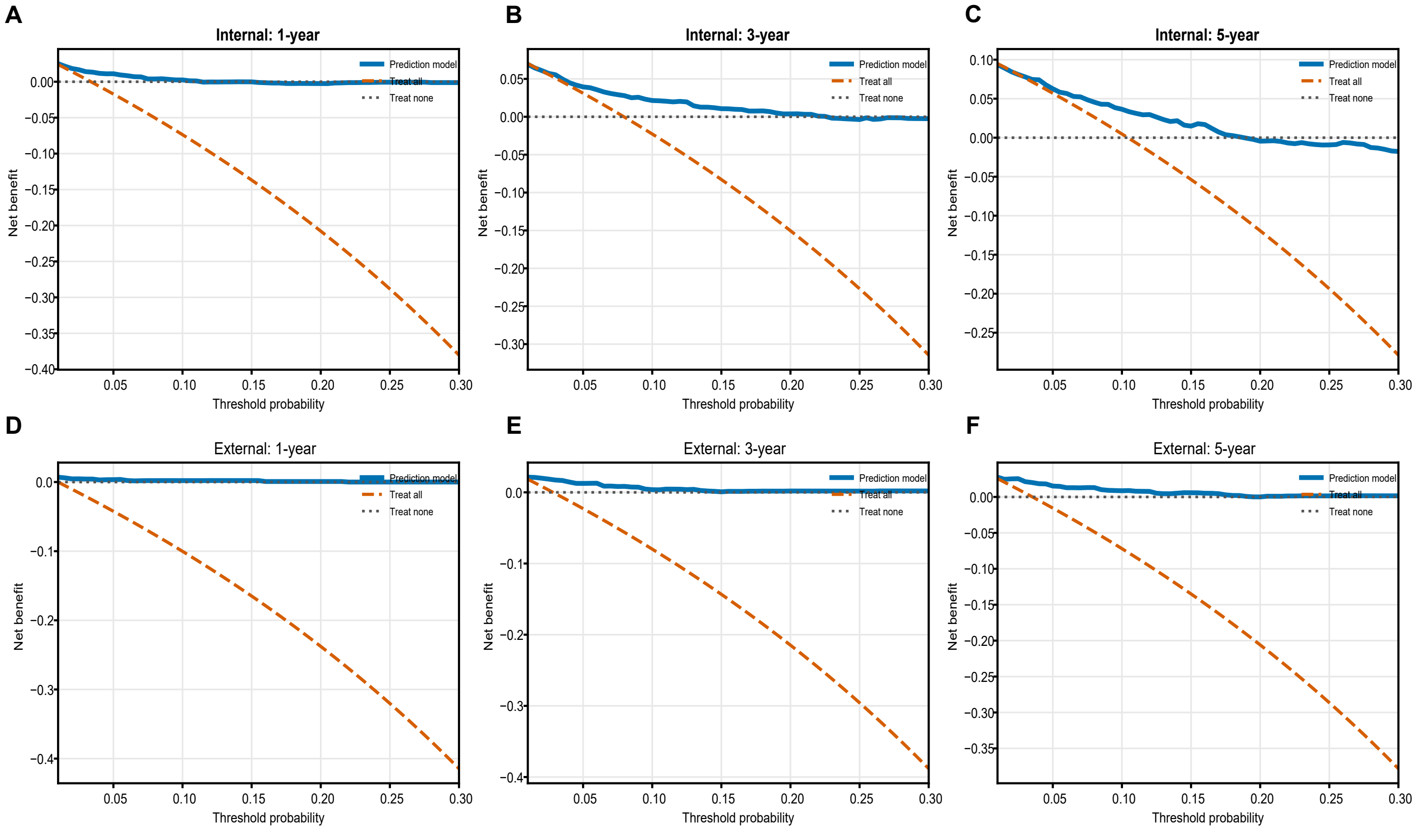

Figure S11. Decision curve analysis (DCA) of the nomogram for predicting cancer-specific survival (CSS) in early gastric cancer. (A–C) DCA for 1-, 3-, and 5-year CSS in the internal validation cohort (A, 1-year; B, 3-year; C, 5-year), showing net benefit across threshold probabilities. (D–F) DCA for 1-, 3-, and 5-year CSS in the external validation cohort (D, 1-year; E, 3-year; F, 5-year). CSS, cancer-specific survival; DCA, decision curve analysis.
